# A MFS-like plasma membrane transporter required for *Leishmania* virulence protects the parasites from iron toxicity

**DOI:** 10.1371/journal.ppat.1007140

**Published:** 2018-06-15

**Authors:** Maria Fernanda Laranjeira-Silva, Wanpeng Wang, Tamika K. Samuel, Fernando Y. Maeda, Vladimir Michailowsky, Iqbal Hamza, Zhongchi Liu, Norma W. Andrews

**Affiliations:** 1 Department of Cell Biology and Molecular Genetics, University of Maryland, College Park, Maryland, United States of America; 2 Department of Animal and Avian Sciences, University of Maryland, College Park, Maryland, United States of America; 3 Faculdade de Medicina, Setor Parasitologia, Universidade Federal do Ceará, Fortaleza, Ceará, Brazil; London School of Hygiene & Tropical Medicine, UNITED STATES

## Abstract

Iron is essential for many cellular processes, but can generate highly toxic hydroxyl radicals in the presence of oxygen. Therefore, intracellular iron accumulation must be tightly regulated, by balancing uptake with storage or export. Iron uptake in *Leishmania* is mediated by the coordinated action of two plasma membrane proteins, the ferric iron reductase LFR1 and the ferrous iron transporter LIT1. However, how these parasites regulate their cytosolic iron concentration to prevent toxicity remains unknown. Here we characterize *Leishmania* Iron Regulator 1 (LIR1), an iron responsive protein with similarity to membrane transporters of the major facilitator superfamily (MFS) and plant nodulin-like proteins. LIR1 localizes on the plasma membrane of *L*. *amazonensis* promastigotes and intracellular amastigotes. After heterologous expression in *Arabidopsis thaliana*, LIR1 decreases the iron content of leaves and worsens the chlorotic phenotype of plants lacking the iron importer IRT1. Consistent with a role in iron efflux, LIR1 deficiency does not affect iron uptake by *L*. *amazonensis* but significantly increases the amount of iron retained intracellularly in the parasites. LIR1 null parasites are more sensitive to iron toxicity and have drastically impaired infectivity, phenotypes that are reversed by *LIR1* complementation. We conclude that LIR1 functions as a plasma membrane iron exporter with a critical role in maintaining iron homeostasis and promoting infectivity in *L*. *amazonensis*.

## Author summary

Leishmaniasis is a human infectious disease that is endemic in many tropical and subtropical areas of the world. It is caused by transmission of the protozoan parasite *Leishmania* through bites of infected sand flies. The symptoms of leishmaniasis range from disfiguring skin lesions to potentially deadly visceralizing disease, and close to 1 billion people live in areas that place them at risk of infection. There is no vaccine available and the drugs currently used to treat this disease are highly toxic and/or expensive, with parasite resistance to these drugs also on the rise. Iron is an essential micronutrient for *Leishmania*, and the parasite’s ability to balance their intracellular iron levels is crucial for their survival and replication inside host cells. Previous studies identified proteins involved in iron uptake that are essential for *Leishmania* infectivity. In this study we identify LIR1, a protein that mediates iron export and therefore prevents iron from accumulating to toxic levels inside the parasites. Absence of LIR1 increases the sensitivity of *Leishmania* to iron toxicity and abolishes parasite infectivity. Given the critical role of LIR1 in iron homeostasis and its importance for parasite virulence, LIR1 is a promising new target for leishmaniasis therapeutic intervention.

## Introduction

*Leishmania spp* are intracellular protozoan parasites that cause human leishmaniasis, a disease spectrum that can vary from self-healing cutaneous lesions to lethal visceralizing disease. *Leishmania* is endemic in about 90 countries throughout the world, and has been spreading from rural to urban areas in recent years [1–4]. Currently, an estimated 12 million people are infected with *Leishmania* and close to 1 billion people may be at risk (WHO) [5,6]. There is no established vaccine and the treatments available can be highly toxic, expensive and/or of limited effectiveness, making the identification of new drug targets an urgent need.

Iron is a critical cofactor for many conserved metabolic pathways in cells. However, free iron can be cytotoxic, because of its ability to generate reactive hydroxyl radicals via the Fenton reaction [7,8]. Thus, iron uptake into cells must be balanced with mechanisms for iron sequestration, storage, and/or export. A typical cellular strategy for storage of intracellular iron is the formation of complexes within ferritin, a widely expressed cytosolic protein that binds and releases iron in a controlled fashion [9,10].

Iron is also an essential micronutrient for *Leishmania*, and restriction in iron availability is a demonstrated host defense mechanism against these parasites [11,12].

Previous studies showed that iron acquisition in *Leishmania* is mediated by LIT1 (*Leishmania* Iron Transporter 1) and LFR1 (*Leishmania* Ferric Reductase 1), transmembrane proteins that have homology with plant IRT1 (iron-regulated transporter 1) and plant FRO1 (ferric oxidoreductase 1), respectively [13,14]. LFR1 reduces Fe^3+^ to Fe^2+^, the soluble form of iron that is translocated across the parasite’s plasma membrane by LIT1. The *Leishmania* genome also encodes a frataxin-like protein that may function in iron storage inside the mitochondria [15], but there are no genes encoding cytosolic proteins with similarity to ferritin. This raises an important question: How do these parasites prevent accumulation of free iron in the cytosol to avoid toxicity?

In addition to storage in association with a ferritin-like protein, yeast cells utilize the CCC1 membrane transporter to translocate iron from the cytosol into vacuoles [16]. In plants, sequestration of cytosolic iron in vacuoles is mediated by vacuolar iron transporters (VITs) that are homologous to CCC1 [17]. Plants also express nodulin-like membrane proteins, which share sequence similarity with VIT1 and yeast CCC1 transporters [18]. Nodulin-like proteins appear to be involved in the transmembrane transport of iron and other compounds [19], but their precise functional role in plant iron metabolism remains to be determined. Given that the *L*. *amazonensis* ferrous iron importer LIT1 [13] and the ferric iron reductase LFR1 [14] have orthologs in plants [15], we set out to investigate whether parasite nodulin-like proteins might also be involved in iron homeostasis.

In this study we identified and characterized the *Leishmania* Iron Regulator 1 (LIR1), a plasma membrane protein with similarity to MFS (Major Facilitator Superfamily) membrane transporters [20–22] and that contains a nodulin-like domain. Our results indicate that *L*. *amazonensis* LIR1 functions in iron export, revealing a mechanism by which the parasites can avoid toxicity by preventing excess intracellular iron accumulation. In agreement with an important role in iron homeostasis, we also demonstrate that LIR1 is essential for *L*. *amazonensis* intracellular replication in macrophages and for the development of pathology in mice.

## Results

### *Leishmania* encodes a MFS-type plasma membrane protein that is upregulated in response to excess extracellular iron

Analysis of the previously published whole-genome transcriptome profile of *L*. *amazonensis* genes modulated by iron deprivation [23] identified LmxM.21.1580 as an iron-responsive gene. This gene, designated *Leishmania Iron Regulator 1* (*LIR1*) (Genbank ID KY643495), is conserved among all *Leishmania* species represented in genome databases. Potential orthologs were also found in *Crithidia fasciculata* (TriTrypDB CFAC1_130025500), *Endotrypanum monterogeii* (TriTrypDB EMOLV88_210023200), *Leptomonas pyrrhocoris* (TriTrypDB LpyrH10_14_2060), *Leptomonas seymouri* (TriTrypDB Lsey_0232_0200), *Trypanosoma cruzi* (TriTrypDB TcCLB.402857.10) and *Trypanosoma brucei* (TriTrypDB Tb927.7.5940) [24].

**Fig 1:**
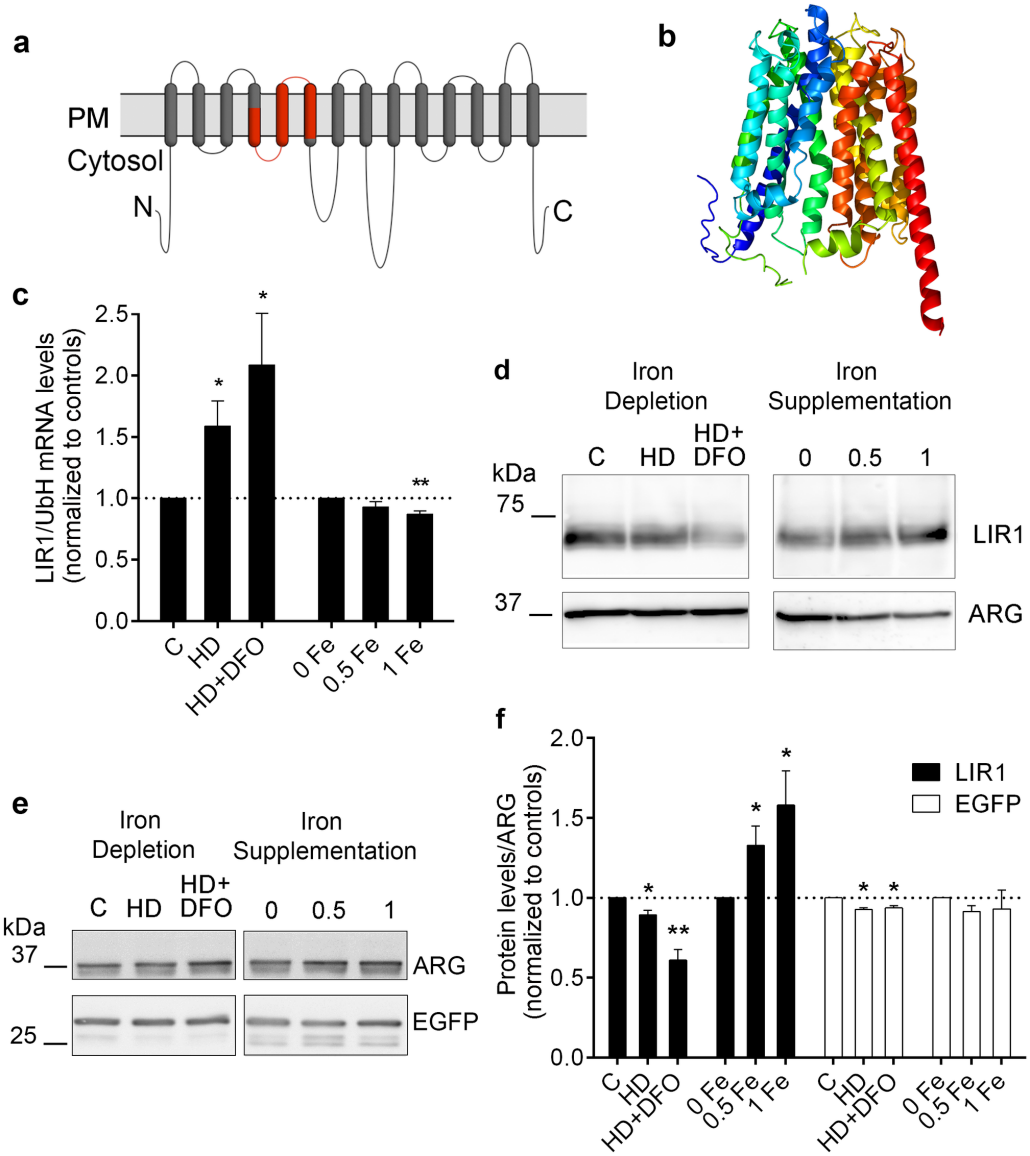
LIR1 is a *Leishmania* iron responsive multi-pass MFS-type protein. (a) Membrane topology model for LIR1, illustrating the 14 predicted transmembrane domains. Highlighted in red is the region with similarity to plant nodulin-like proteins. PM, plasma membrane. N, amino-terminus. C, carboxy-terminus. (b) 3D-structure model of LIR1 generated with the Phyre2 software [25]. (c) Endogenous *LIR1* transcripts were quantified and normalized by *UbH* transcript levels in *L*. *amazonensis* grown in regular promastigote medium (C, control) or under conditions of iron depletion (HD: heme depletion; HD+DFO: heme depletion plus 50 μM DFO) or iron supplementation (0.5 Fe, 0.5 mM FeSO_4_; 1 Fe, 1 mM FeSO_4_). The data represent the mean ± SEM of *LIR1*/*UbH* transcript levels normalized to their respective controls in 4 (iron depletion) or 3 (iron supplementation) independent experiments. * p = 0.028 (HD vs C); * p = 0.0423 (HD+DFO vs C); ** p = 0.0085 (1 Fe vs 0 Fe). (d,e) LIR1 (d) or EGFP (e) protein levels determined by Western blot in total lysates of promastigotes ectopically expressing GFP-LIR1 and grown under iron depletion or iron supplementation. GFP-LIR1 and EGFP were detected with anti-GFP antibodies, and anti-arginase (ARG) antibodies were used as loading control. (f) LIR1 and EGFP Western blots were quantified by densitometry and normalized using ARG protein levels. The graph shows the mean ± SEM of LIR1/ARG and EGFP/ARG normalized by their respective controls in 3 independent experiments. For LIR1: * p = 0.02 (HD vs C); ** p = 0.004 (HD+DFO vs C); * p = 0.05 (0.5 and 1 Fe vs 0 Fe). For EGFP: * p = 0.01 (HD and HD+DFO vs C).

*LIR1* encodes a 661 amino acid protein with a predicted molecular mass of 73 kDa and 14 putative transmembrane domains (Fig 1a). A 100% confidence alignment of LIR1 with a template for MFS general substrate transporters was obtained through 3D structure modeling using the Phyre2 software [25] (Fig 1b). Conserved domain analysis (NCBI Conserved Domains Database) revealed domains similar to plant nodulin-like proteins [18,19] (Bit Score 39.63; E-value: 1.31e-03) and MFS-type small solute membrane transporters [20–22] (Bit Score 37.29; E-value: 9.98e-03).

Quantification of endogenous *LIR1* mRNA revealed upregulation after promastigotes were cultured for 18–24 h in heme-depleted medium (HD), heme-depleted medium containing the iron chelator deferoxamine (HD+DFO) or medium depleted in iron using Chelex (ID) (Fig 1c, S1a Fig). Conversely, downregulation was observed after 4 days of culture in iron-supplemented medium (Fig 1c, 1mM FeSO_4_). No detectable upregulation above the already high levels observed for ectopically expressed *LIR1* transcripts was observed after 18 h of culture in iron depleted medium (HD+DFO), but a significant downregulation of ectopically expressed *LIR1* transcripts also occurred after 4 days of culture in iron supplemented medium (S1b Fig). However, the unusual polycystronic mode of gene transcription in trypanosomatids [26] does not allow direct transcript-protein extrapolations, because gene expression is regulated largely at the post-transcriptional level [27]. Thus, we used antibodies to quantify ectopically expressed (GFP-tagged) LIR1 proteins in *L*. *amazonensis* promastigotes exposed to changes in iron availability (Fig 1d–1f). Remarkably, after 18 h of incubation in heme-depleted medium containing the iron chelator DFO (HD+DFO), GFP-LIR1 protein levels were markedly decreased, an opposite effect to what was observed for the mRNA under the same conditions. Conversely, when the parasites were cultured for 4 days in medium supplemented with iron sulfate, there was a dose-dependent increase in the amount of GFP-LIR1 protein detected (Fig 1d and 1f). This effect was specific for GFP-LIR1, as iron depletion caused much smaller changes and iron supplementation caused no significant changes in ectopically expressed cytosolic EGFP (Fig 1e and 1f). These LIR1 results add to previous evidence indicating a lack of correlation between transcript and protein levels in *Leishmania* [27]. Considering that *LIR1* untranslated regions were not included in the GFP-LIR1 expression constructs, our results suggest the potential existence of mechanisms for iron-dependent regulation of LIR1 expression at the protein turn-over level. However, in the absence of a specific anti-LIR1 antibody (which is challenging to generate for such a large and multiple membrane spanning domain protein), our current results do not rule out other regulatory mechanisms at the level of translation or mRNA stability.

**Fig 2:**
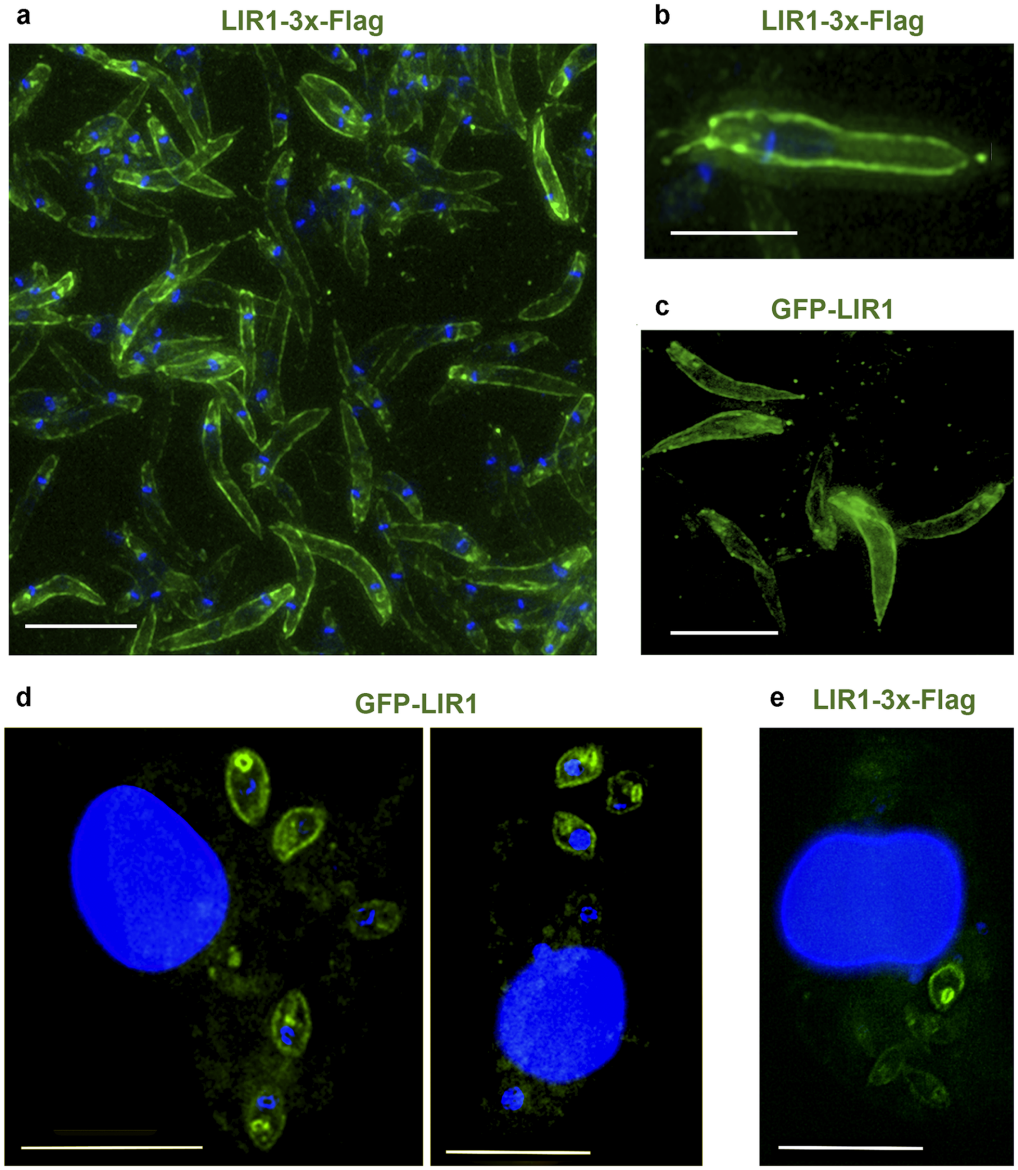
LIR1 localizes at the plasma membrane of promastigotes and intracellular amastigotes. (a,b) Immunofluorescence deconvolution images of *L*. *amazonensis* promastigotes expressing LIR1 with a 3xFlag at the C-terminus. Blue, DAPI DNA staining. Green, anti-3xFlag antibodies. Scale bars: 7 μm (a) and 3.5 μm (b). (c) Deconvolution fluorescence microscopy image of *L*. *amazonensis* promastigotes expressing LIR1 fused to GFP at the N-terminus. Scale bar: 11 μm. (d,e) Immunofluorescence deconvolution images of BMM infected for 48 h with axenic amastigotes expressing LIR1 fused to GFP at the N-terminus (d) or 3xFlag at the C-terminus (e). Blue, DAPI DNA staining. Green, GFP-LIR1 or anti-3xFlag antibodies. Scale bars: 11 μm.

Immunofluorescence of *L*. *amazonensis* promastigotes (insect life cycle stages) expressing the plasmids p-LIR1-3xFlag (Fig 2a and 2b) and p-GFP-LIR1 (Fig 2c) revealed that both tagged forms of LIR1 (3xFlag on the C-terminus or GFP on the N-terminus) are targeted to the parasite’s plasma membrane. Similar plasma membrane localization was detected in the mammalian intracellular amastigote stages, after mouse bone marrow macrophages (BMM) were infected with *L*. *amazonensis* expressing p-GFP-LIR1 (Fig 2d) or p-LIR1-3xFlag (Fig 2e). The bright fluorescent circular structures observed in intracellular amastigotes, seen with both GFP and 3xFlag tags (Fig 2d and 2e), likely reflect the parasite’s flagellar pocket, a membrane invagination that is continuous with the plasma membrane [28,29]. Thus, our findings identify LIR1 as a plasma membrane protein with similarity to MFS transmembrane transporters that is downregulated during iron deprivation and upregulated when the parasites are exposed to excess iron. This increase in protein expression under excess iron and decrease under low iron suggested a role for LIR1 in managing the size of the parasite’s intracellular iron pool.

### LIR1 expressed in *Arabidopsis thaliana* is targeted to the plasma membrane and decreases the iron content of plant tissues

**Fig 3:**
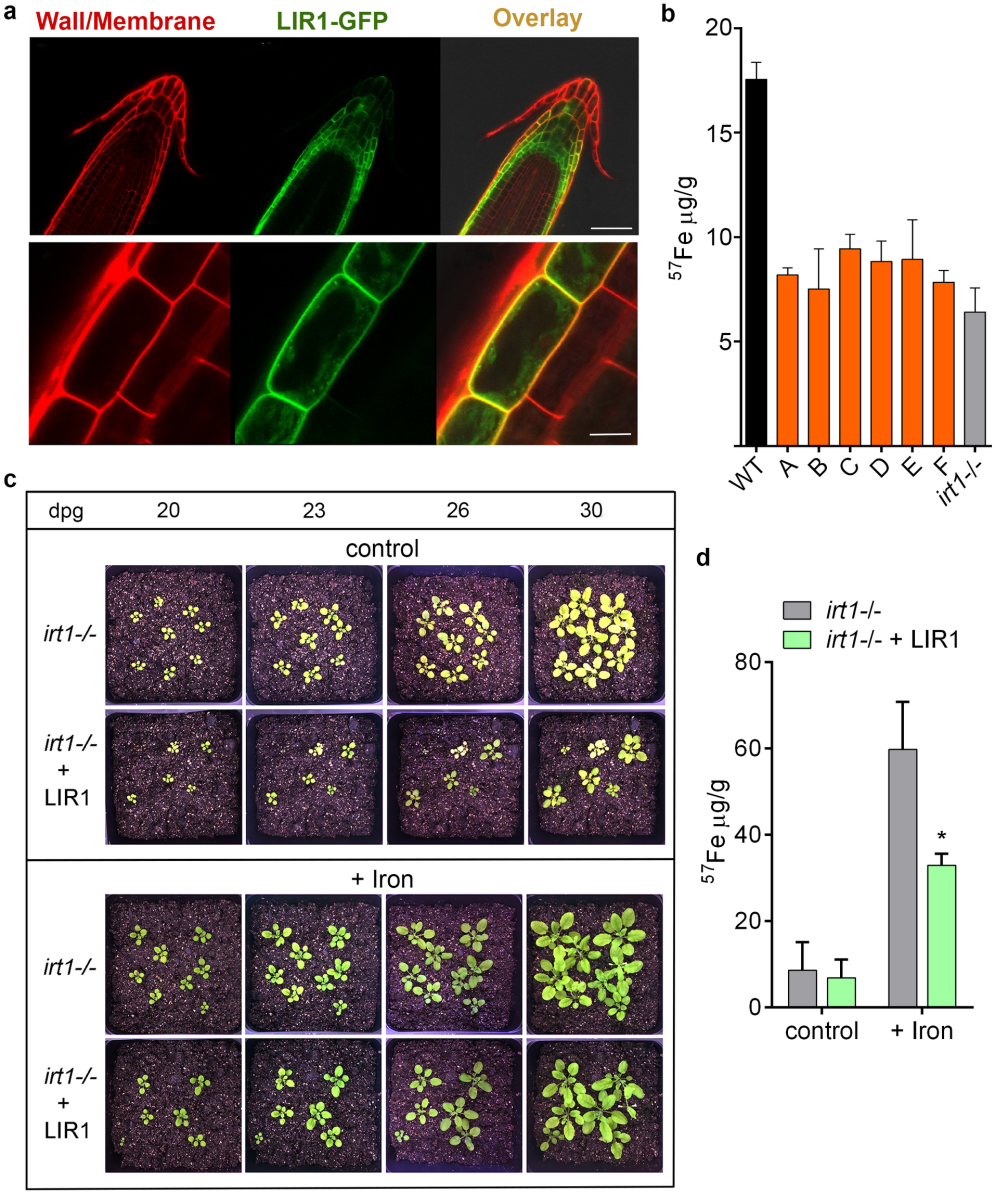
LIR1 expressed in *Arabidopsis thaliana* is targeted to the plasma membrane and decreases the iron content of leaves. (a) Confocal images of root sections of *Arabidopsis thaliana* expressing LIR1 fused to GFP at the C-terminus. Red, cell wall/plasma membrane staining with propidium iodide (PI). Green, LIR1-GFP. Scale bars: 50 μm (upper panel) and 10 μm (lower panel). (b) ICP-MS quantification of the iron total content from leaves of wild type (WT), WT expressing LIR1, and *irt1-/- Arabidopsis thaliana*. A-C, independent *Arabidopsis* transgenic lines containing the 35S promoter-driven *LIR1* cDNA (35S::LIR1). D-F, independent transgenic lines of 35S::LIR1+GFP (35S promoter-driven LIR1 cDNA fused to GFP). The data represent the mean ± SD of 3 samples for each strain, normalized for tissue weight. (c) Phenotype of *irt1-/-* and *irt1-/-* expressing LIR1 (*irt1-/-* + LIR1) *A*. *thaliana* plants grown in soil irrigated with either water (Control) or 0.5 g/L Sequestrene (+ Iron) for 20, 23, 26 and 30 days post germination (dpg). (d) ICP-MS quantification of the iron total content from leaves of *irt1-/-* and *irt1-/-* expressing LIR1 (*irt1-/-* + LIR1) *A*. *thaliana* irrigated with either water (control) or 0.5 g/L Sequestrene (+ Iron). Plant tissue was collected 30 days post germination (dpg). The data represent the mean ± SD of 3 leave samples for each strain, normalized for tissue weight. * p = 0.0147 (*irt1-/-* + Iron vs. *irt1-/-* + LIR1 + Iron).

Given the sequence similarity of *LIR1* with plant nodulin-like proteins, we investigated the molecular function of LIR1 in a heterologous system by generating transgenic *Arabidopsis thaliana* plants expressing *LIR1*. As observed in *L*. *amazonensis*, LIR1-GFP fusion constructs were predominantly targeted to the plasma membrane in *A*. *thaliana* tissues, as shown in root cells (Fig 3a) and in leaf mesophyll protoplasts (S2a Fig). Remarkably, when the total iron content of leaves in plants expressing *LIR1* was quantified by inductively coupled plasma mass spectrometry (ICP-MS), a marked decrease was observed in relation to wild type (WT), reaching levels similar to what is observed in leaves of *irt1-/-* plants that lack the major iron uptake transporter IRT1 [30,31] (Fig 3b). This result directly indicated a function for LIR1 in iron export.

WT plants expressing *LIR1* did not show the severe growth defect and chlorotic phenotype that is typical of the iron deficient *irt1-/- A*. *thaliana* mutants [30,31] (S2b Fig), possibly as a result of compensatory mechanisms (in addition to iron the IRT1 transporter also mediates the uptake of zinc, manganese and cobalt by plant roots [31]). To further address this issue and to directly determine whether *LIR1* had an impact on the iron-deficiency phenotype of *irt1-/- A*. *thaliana*, we generated transgenic *irt1-/-* plant lines expressing *LIR1*. These transgenic plants presented a markedly worsened chlorotic phenotype and had a more severe growth defect, when compared to the *irt1-/-* line not expressing *LIR1* (Fig 3c, top panels). Under normal growth conditions ICP-MS analysis did not detect a statistically significant reduction in the iron content of the *irt1-/-* lines expressing *LIR1*, possibly due to the already critically low iron content of this line. However, when the iron supplement Sequestrene [31] was supplied at 0.5 g/L, a marked reduction in iron content was evident in *irt1-/-* lines expressing *LIR1*, when compared to plants not expressing LIR1 (Fig 3d). Confirming the link between these phenotypes and the regulation of iron homeostasis, addition of Sequestrene reversed the chlorotic and growth phenotypes of both strains (Fig 3c, bottom panels). Thus, the results of these heterologous expression experiments in *A*. *thaliana* demonstrate a role for LIR1 in reducing the size of the intracellular iron pool by directly promoting iron export across the plasma membrane.

### LIR1 regulates intracellular iron levels in *Leishmania*, exporting iron to prevent its intracellular accumulation

To assess the functional role of LIR1 in *L*. *amazonensis* we generated heterozygote *LIR1/lir1*^*-*^ (SKO) and homozygote null *lir1*^*-*^*/lir1*^*-*^ (KO) clones by transfecting promastigotes with gene deletion constructs, followed by drug selection (S3a Fig). To restore *LIR1* expression, the *LIR1* ORF sequence was stably integrated into the SSU rRNA locus of the KO mutant, yielding the add-back line *lir1*^*-*^*/LIR1* (AB) (S3b Fig). Independent SKO, KO and AB clones were isolated and analyzed in parallel. The loss of *LIR1*, replacement of both *LIR1* alleles and integration of *LIR1* into the SSU rRNA locus were confirmed by PCR analysis (S3c and S3d Fig).

**Fig 4:**
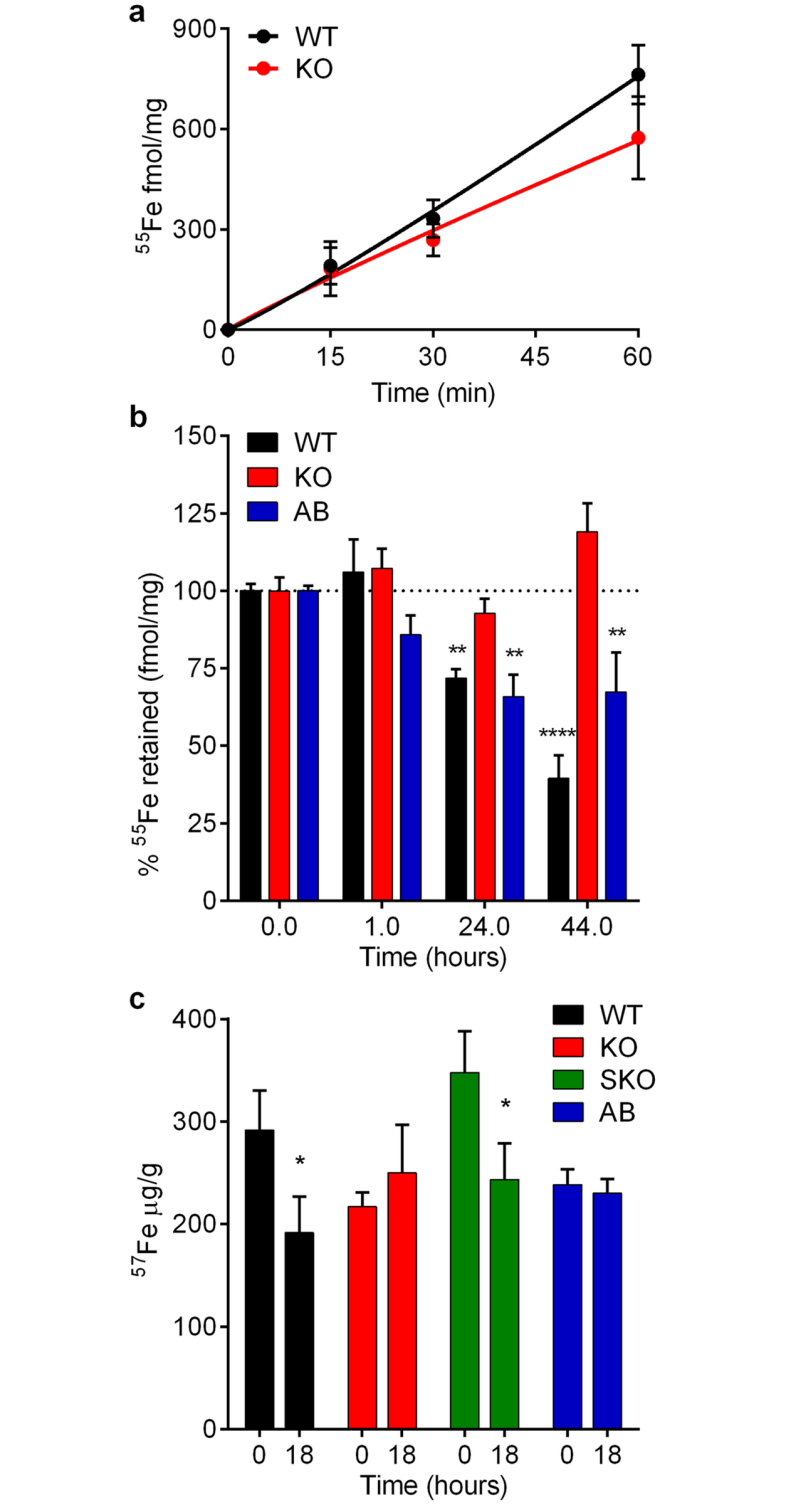
LIR1 expression mediates iron efflux and prevents accumulation of iron and other transition metals in *Leishmania*. (a) Time course of iron uptake in wild type (WT) and *LIR1* double knockout (KO) *L*. *amazonensis* promastigotes incubated with ^55^Fe for 60 min and analyzed in triplicate. Each data point represents the mean ± SD from 3 independent experiments performed in triplicate and expressed as fmol/mg of total cell protein. (b) Iron efflux assessed by quantifying the intracellular ^55^Fe pool after incubating wild type (WT), *LIR1* double knockout (KO) and add-back (AB) *L*. *amazonensis* promastigotes with ^55^Fe for 90 min followed by a chase for 1, 24 and 44 h in buffer lacking ^55^Fe and containing excess cold iron. The data are shown as percentage of ^55^Fe retained in relation to time zero. Each data point represents the mean ± SEM of 3 independent experiments performed in triplicate and expressed as fmol/mg of total cell protein. ** p = 0.0014 (WT vs. KO 24 h); ** p = 0.0056 (AB vs. KO 24 h); ** p = 0.0059 (AB vs. KO 44 h); **** p < 0.0001 (WT vs. KO 44 h). (c) ICP-MS determination of the total iron content of wild type (WT), *LIR1* double knockout (KO), *LIR1* single knockout (SKO) and add-back (AB) *L*. *amazonensis* promastigotes immediately after being washed (0 h) or after incubation in fresh regular growth medium for 18 h. ICP-MS values were normalized by the total protein content of the samples. The data represent the mean ± SD of 3 independent experiments, each point analyzed in duplicate. * p = 0.0294 (WT 0 vs. 18 h) and p = 0.284 (SKO 0 vs. 18 h).

We directly assessed the role of LIR1 in iron transport by comparing the ^55^Fe uptake profile of WT and LIR1 KO mutants. Lack of LIR1 expression in the KO strain did not significantly change ^55^Fe uptake in relation to WT (Fig 4a), reinforcing the conclusion that LIR1 does not function as an iron importer. To examine a potential iron-export phenotype, WT, LIR1 KO and AB cells under iron starvation were pulse-labeled with ^55^Fe for 90 min and then analyzed after specific chase periods for the amount of ^55^Fe retained (Fig 4b). While the intracellular amount of ^55^Fe decreased progressively over time in WT parasites, this was not observed with LIR1 KO parasites, which showed a significant impairment in iron efflux. Importantly, recovery of this phenotype was observed with AB parasites after 24 h of chase, supporting the conclusion that LIR1 promotes iron efflux.

We also quantified the total amount of parasite-associated iron by inductively coupled plasma mass spectrometry (ICP-MS) before (promastigotes taken from a late log culture, washed and lysed immediately) or after 18 h of incubation in fresh growth medium (which is expected to contain higher iron levels). We found that WT *Leishmania* promastigotes responded to transfer to fresh media by decreasing their intracellular iron content within the 18 h period. Under the same conditions, no decrease in the total iron content of LIR1 KO parasites was observed, suggesting that loss of LIR1 impairs the parasites’ ability to regulate their total intracellular iron levels (Fig 4c). The SKO line showed a decrease in iron content similar to WT, suggesting that one *LIR1* allele was sufficient to reduce intracellular iron levels under the assay conditions. A statistically significant difference was not observed between the 0 and 18 h time points with the AB line in this assay (Fig 4c), possibly due to abnormal regulation of the intracellular iron pool of parasites ectopically expressing LIR1. A failure to fully restore WT phenotypes is a common occurrence when ectopically expressing genes in *Leishmania*, because expression levels cannot be tightly regulated [32–34]. Interestingly, however, reversal of the KO phenotype was observed with the AB line in ^55^Fe efflux assays (Fig 4b), suggesting that LIR1 overexpression is still able to restore the dynamics of the cytoplasmic labile iron pool that is readily mobilized by plasma membrane transporters, but may have long term deleterious effects on the total iron content of cells (detected by ICP-MS), which includes iron metabolically complexed to other molecules and stored inside various organelles. Collectively, these results further strengthen the evidence that LIR1 functions as a plasma membrane transporter that promotes iron export.

### LIR1 prevents iron toxicity during *Leishmania* replication

**Fig 5:**
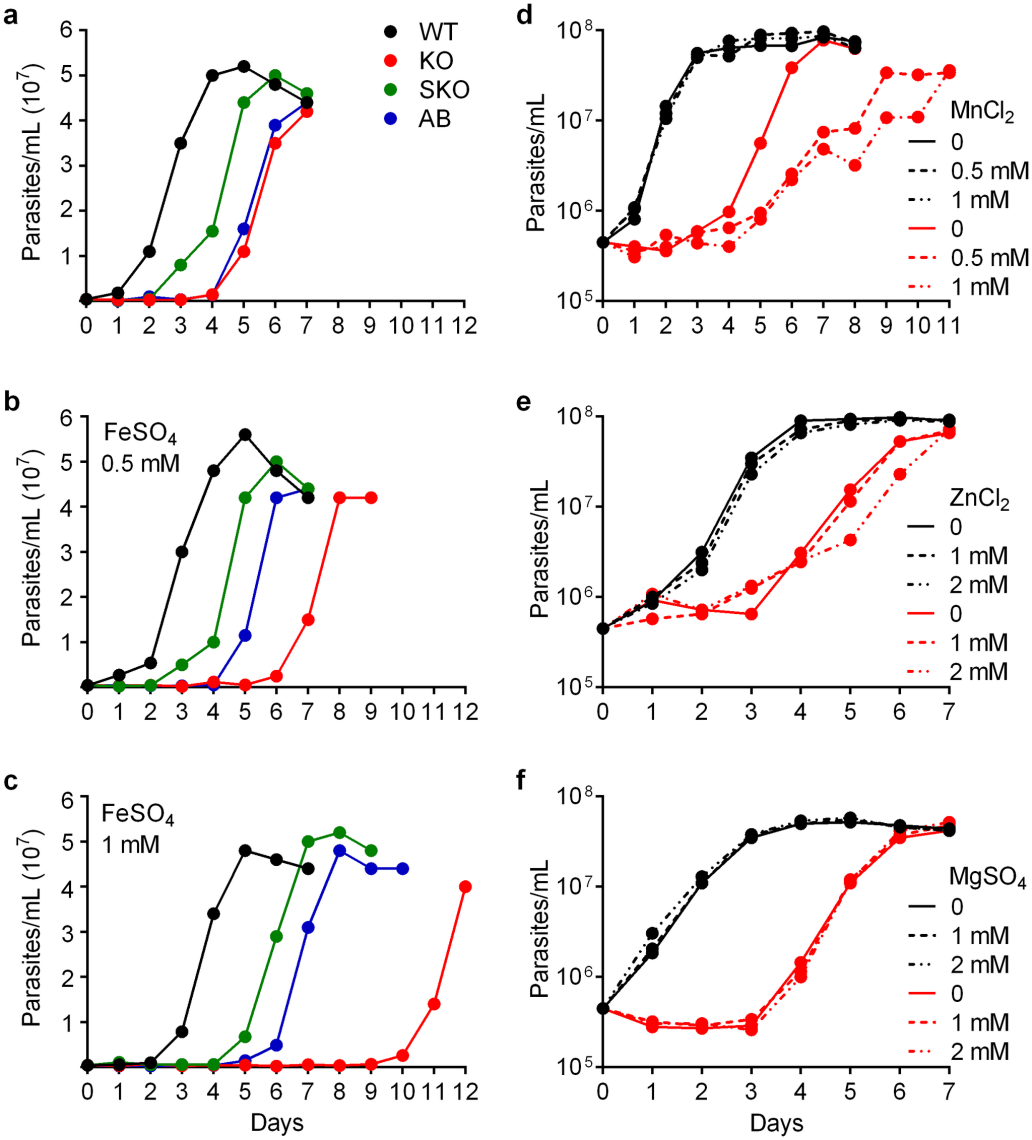
LIR1 deficiency enhances the toxic effect of excess iron and other transition metals during promastigote replication. (a-c) Growth curves of wild type (WT), *LIR1* single knockout (SKO), *LIR1* double knockout (KO) and add-back (AB) *L*. *amazonensis* promastigotes in regular growth medium (a) or in the same medium containing extra iron: 0.5 mM (b) and 1 mM (c) FeSO_4_. The values correspond to the average of triplicate determinations, in one experiment that is representative of 3 independent experiments (see also supplementary S4 Fig). (d-f) Growth curves of wild type (WT) and *LIR1* double knockout (KO) promastigotes in regular growth medium (continuous lines) or in the same medium supplemented with (dotted lines) (d) 0.5 and 1 mM MnCl_2_, (e) 1 and 2 mM ZnCl_2,_ (f) 1 and 2 mM MgSO_4_. The values correspond to the average of triplicate determinations, in one experiment that is representative of 2 independent experiments.

Quantification of *in vitro* promastigote growth showed that *LIR1* deficiency causes a marked delay on the parasites’ ability to initiate replication (Fig 5a, S4b and S4c Fig). KO parasites showed a 3-day growth delay in relation to wild type (WT), while SKO had an intermediate phenotype. Notably, addition of extra iron to the culture media in the form of 0.5 mM FeSO_4_ worsened the growth delay phenotype of the KO, without similarly affecting growth of the SKO and AB lines (Fig 5b). Addition of 1 mM FeSO_4_ further worsened the KO growth delay, while causing a markedly smaller effect on the WT, SKO and AB lines (Fig 5c). Interestingly, the mutant parasite strains were able to grow at rates similar to the WT strain after the several-day delay periods. This pattern suggests the existence of late-acting mechanisms that allow promastigotes to overcome iron sensitivity and replicate in liquid culture. A similar deleterious effect of iron supplementation on promastigote replication was seen in several independent experiments (S4a Fig). These results suggest that the LIR1 transporter can protect *Leishmania* promastigotes from the toxic effects of excessive intracellular iron.

Many membrane transporters that have iron as a substrate also translocate other transition metals, most commonly manganese and at a lesser extent zinc and copper [35]. In agreement with these findings, increasing the concentration of manganese in the culture medium also worsened the KO line growth delay phenotype without affecting WT promastigotes (Fig 5d). A less pronounced but still detectable inhibitory effect on the replication of KO parasites was observed in the presence of elevated concentrations of zinc (Fig 5e). The toxic effects of excess manganese and zinc on LIR1 KO were, however, significantly less pronounced than what was observed with iron (Fig 5a–5c). In contrast, addition of the non-transition metal magnesium at similar concentrations had no effect on the WT or KO line growth pattern (Fig 5f). Taken together, our results indicate that LIR1 functions as a plasma membrane transition metal transporter that can protect *Leishmania* from the toxic effects of high concentrations of iron, and of manganese and zinc to a smaller extent.

### LIR1 is required for *Leishmania* infectivity

**Fig 6:**
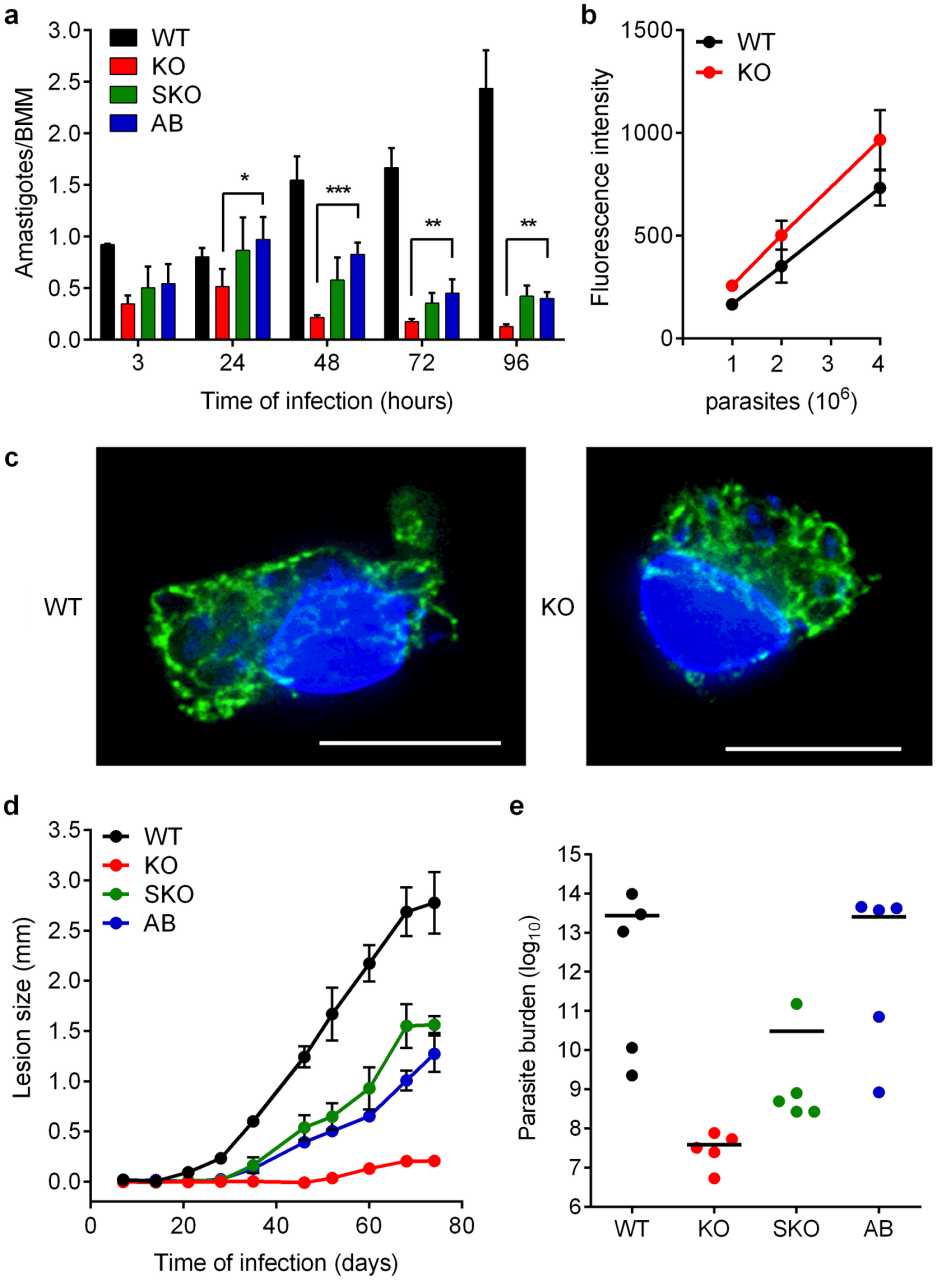
LIR1 deficiency markedly reduces *L*. *amazonensis* virulence. (a) Microscopic quantification of intracellular parasites in BMM infected with purified metacyclic forms of wild type (WT), *LIR1* double knockout (KO), *LIR1* single knockout (SKO) and add-back (AB) *L*. *amazonensis* (multiplicity of infection = 3) for 3, 24, 48, 72 and 96 h. The values represent the mean ± SD of triplicate determinations in one experiment that is representative of 3 independent experiments. * p = 0.031 (KO vs. AB 24 h), ** p = 0.0063 (KO vs. AB 72 h); p = 0.0019 (KO vs. AB 96 h); *** p = 0.0007 (KO vs. AB 48 h). (b) AlamarBlue fluorescence viability assay of 10^6^, 2x10^6^ and 4x10^6^ purified metacyclics purified from WT and KO promastigote cultures. The values represent the mean ± SD of 3 independent experiments. (c) Immunofluorescence deconvolution images of BMM infected for 3 h with WT and KO metacyclic promastigotes. Blue, DAPI DNA staining; green, lysosomes and PV membranes stained with anti-Lamp-1 antibodies. Scale bars = 11 μm. (d) Cutaneous lesion development in C57BL/6 mice inoculated with 10^6^ purified metacyclic forms of wild type (WT), *LIR1* double knockout (KO), *LIR1* single knockout (SKO) and add-back (AB) *L*. *amazonensis*. The data represent the mean ± SEM of measurements from 5 different mice in each group. (e) Parasite load in footpad tissues determined at 10.5 weeks after infection (n = 5).

Infection of mouse bone marrow macrophages (BMM) revealed that partial *LIR1* deficiency (SKO) impairs the sustained intracellular replication that is observed with WT *L*. *amazonensis*, while complete deficiency (KO) leads to no replication and a strong reduction in the number of intracellular parasites over time (Fig 6a). The metacyclic life cycle stages of *L*. *amazonensis* used to infect the BMM were purified after selective agglutination of promastigotes with the 3A.1 mAb antibody [36] and tested in viability assays. These tests showed that *LIR1* KO metacyclics were as viable as WT metacyclics prior to macrophage infection (Fig 6b), and there were also no differences in the ability of WT or KO parasites to induce formation of the enlarged parasitophorous vacuoles (PV) that are typical of *L*. *amazonensis* [37] in macrophages (Fig 6c). Thus, we conclude that the marked inability to survive and replicate intracellularly observed in *LIR1* KO parasites is not related to metabolic defects in the metacyclic population used to initiate the infection. Rather, our results indicate the LIR1 expression is required for the parasites to survive and replicate as intracellular amastigotes within macrophage PVs. Strengthening this conclusion, the number of intracellular parasites of the genetically complemented AB line was significantly higher than the KO line at all time points subsequent to the initial 3 h (Fig 6a). Similar results were observed in macrophage infection experiments performed with independent SKO, KO and AB clones (S5a and S5b Fig).

To determine the importance of LIR1 for the ability of *L*. *amazonensis* to generate cutaneous lesions *in vivo*, purified metacyclic forms were inoculated into footpads of C57BL/6 mice. As expected, all mice inoculated with WT parasites developed progressive cutaneous lesions within 3–4 weeks (Fig 6d). In contrast, lesion development in mice injected with KO parasites was markedly reduced, with slight footpad swelling detected only between days 49 and 73 (after which all mice were sacrificed because WT lesions reached the maximum size allowed by our animal welfare protocol). Inoculation with SKO and AB parasites led to intermediate-size lesions. Parasite load analysis [38] of cutaneous lesions extracted from the infected mice after 73 days revealed 10^6^-fold less parasites in KO-infected mice and 10^3^-fold less in SKO-infected mice, when compared to WT and AB (Fig 6e). Notably, while lesions from mice infected with parasites lacking one *LIR1* allele (SKO) showed intermediate parasite loads, full rescue of the WT phenotype was observed in *LIR1* complemented parasites (AB). Taken together with the results of our experiments following intracellular replication in macrophages (Fig 6a, S5a and S5b Fig), these findings show that LIR1 deficiency prevents the intracellular replication of the vertebrate amastigote forms, not merely delaying their growth as observed with the insect promastigote stages (Fig 5). These findings identify LIR1 as a virulence factor that is required for the successful intracellular replication and cutaneous lesion formation in mammalian hosts by *L*. *amazonensis*.

## Discussion

Despite advances in our understanding of pathways of iron acquisition and metabolism in *Leishmania* [13,14,23,39–42], how these parasites regulate their intracellular labile iron pool to prevent toxicity is still an open question. In this study we clarify this important issue by identifying and characterizing LIR1, to our knowledge the first plasma membrane protein shown to mediate iron export and prevent intracellular iron accumulation in trypanosomatid parasites. First, we found that *LIR1* encodes a predicted multi-pass plasma membrane protein with structural similarity to the MFS group of membrane transporters [20–22]. Second, we showed that expression of LIR1 is regulated by iron, with the protein accumulating in parasites exposed to excess iron and decreasing under iron depletion. Third, when LIR1 is heterologously expressed in *Arabidopsis thaliana* it is targeted to the plasma membrane and decreases the iron content of plant tissues. Finally, we also showed that LIR1 expression in *L*. *amazonensis* regulates the size of the intracellular iron pool, protects the parasites from iron toxicity, and is required for virulence. Collectively, our results indicate that *LIR1* encodes a transmembrane MFS-type protein containing a plant-like nodulin domain that promotes iron export in *Leishmania*.

Our results with *A*. *thaliana* expressing *LIR1* shed light on the potential function of nodulin-like proteins in plants, which were previously linked to mechanisms of iron homeostasis but not directly shown to function as iron exporters [19]. Nodulin-like proteins have significant sequence homology with the plant and yeast vacuolar iron transporters AtVIT1 and ScCCC1, respectively [18]. CCC1 is a membrane transporter that mediates vacuolar iron storage in yeast, protecting yeast cells from iron accumulation in the cytosol [16]. Here we uncovered a similar function but a different subcellular localization for LIR1. While CCC1 is localized on the membrane of yeast vacuoles (lysosome-equivalent organelles), LIR1 is predominantly targeted to the plasma membrane of both life cycle stages of the parasites, the insect promastigotes and the intracellular amastigotes responsible for mammalian infection. Taken together with the regulation of LIR1 protein expression by iron levels in the environment, our findings point to the existence in *Leishmania* of a unique mechanism for managing the intracellular iron pool. Instead of storing iron in the lumen of an intracellular compartment or coupled to cytosolic ferritin, as observed in yeast and other eukaryotes, *Leishmania* parasites may largely rely on directly exporting cytosolic iron to the extracellular environment through the plasma membrane.

Interestingly, the protozoan parasites *Plasmodium falciparum* and *Plasmodium berghei* express a VIT1-like iron transporter that also functions as an iron detoxifier [43], and proteomic studies revealed that *Trypanosoma brucei* expresses a putative VIT1 protein that is targeted to acidocalciosomes and required for parasite growth [44]. However, prior to our study very little additional information was available about the role that nodulin/VIT-like transporters might play in iron homeostasis in protozoan parasites, and their impact on virulence. The data presented here strongly suggests that the nodulin-like *Leishmania* protein LIR1 is directly involved in managing the intracellular iron pool and in preventing iron toxicity. Our findings provide, for the first time, an explanation for how *Leishmania* parasites can maintain iron homeostasis in the absence of ferritin-like cytosolic iron storage proteins. Interestingly, the strongest phenotypes we observed in LIR1-deficient *L*. *amazonensis* involved experiments initiated with metacyclic promastigotes, the infective form that is generated inside sand flies and is responsible for transmission of the infection to mammalian hosts. Our results suggest that excessive accumulation of intracellular iron may be particularly deleterious for metacyclic parasites undergoing reprogramming for differentiation into replicative forms.

Previous studies from our group showed that iron import in *Leishmania* is mediated by LIT1, a plasma membrane ferrous iron transporter [13]. LIT1 transcripts are upregulated in parasites grown in iron depleted media, a response also seen with the plasma membrane proteins LFR1 (ferric iron reductase) [14] and LHR1 (heme transporter) [45]. Interestingly, although the LIR1 mRNA is also upregulated by iron depletion, the opposite was observed at the protein level. This finding is consistent with recent reports that revealed a markedly low (only 20–30%) correlation between mRNA and protein levels in *Leishmania*, as the parasites underwent gene expression changes triggered by the environment. It is noteworthy that in mammalian cells and other higher eukaryotes, expression of genes involved in iron homeostasis is directly controlled by iron regulatory proteins (IRPs) that recognize iron responsive elements (IRE) on mRNAs [46,47]. In contrast, there is no evidence so far for the presence of IREs in *Leishmania* mRNAs. Thus, our results contribute to the emerging view that in the absence of a canonical IRE, regulation of iron importers and exporters may be coupled, opening up an interesting new area for future investigation.

By identifying LIR1 as the first plasma membrane iron exporter in a trypanosomatid parasite, our findings add a critical missing element to the iron homeostasis machinery of this important group of human pathogens. Given the essential role played by LIR1 in *L*. *amazonensis* virulence, its localization on the parasite plasma membrane and the lack of human orthologs, our findings suggest that LIR1 could be a promising target for the future development of therapeutic drugs.

## Materials and methods

### *Leishmania* cultivation

The *L*. *amazonensis* IFLA/BR/67/PH8 strain was provided by Dr. David Sacks (Laboratory of Parasitic Diseases, NIAID, NIH). Promastigote forms were cultured *in vitro* at 26 °C in M199: medium 199 (Gibco, Invitrogen) pH 7.2 supplemented with 10% heat inactivated fetal bovine serum (FBS), 40 mM Hepes, 0.1 mM adenine, 0.0001% biotin, 5 μg/ml hemin (25 mg/ml in 50% triethanolamine), 5 mM L-Glutamine and 5% penicillin-streptomycin. Heme-depleted medium was prepared similarly, omitting hemin addition and replacing regular FBS by heme-depleted FBS. Heme-depleted FBS was generated by treating heat inactivated FBS with 10 mM ascorbic acid for 16 h at room temperature, followed by verification of heme depletion by measuring the optical absorbance at 405 nm [48], 3 rounds of dialysis in cold phosphate-buffered saline (PBS) and filter-sterilization.

Iron-depleted FBS and medium were prepared as previously described [23]. Briefly, iron-depleted FBS was prepared treating 100 ml of heme-depleted FBS with 5 g of Chelex for 3–4 h, followed by filtration to remove Chelex and 4 rounds of dialysis in cold PBS. Iron-depleted medium was prepared similarly to regular M199 without addition of hemin and by replacing regular FBS with iron-depleted FBS. The media was stirred with 5 g/100 ml of Chelex for 1 h at room temperature and filter-sterilized. Ca, Cu, Mn, Mg and Zn ions were added back using the values previously determined by ICP-MS [23]. Promastigote growth curves were initiated with parasites taken from cultures in the stationary phase of growth and resuspended at the indicated densities in fresh growth media containing or not the supplemental metal salts.

### *LIR1* mRNA and protein level quantification

Total RNA was obtained using NucleoSpin RNA kit (Macherey-Nagel GmbH & Co. KG) following manufacturers’ instructions. cDNA was synthesized using SuperScript III Reverse Transcriptase (Invitrogen). For a final reaction volume of 20 μl, 1 μg of total RNA mixed with 0.5 μg oligo(dT)_12-18_ and 1 μl of dNTPs 10 mM was denatured at 65 °C for 5 min; after cooling, 1 μl of DTT 0.1 M, 4 μl of Buffer 5X and 200 U of Reverse Transcriptase were added to the reaction, followed by incubation at 50 °C for 60 min, inactivation at 70 °C for 15 min, and storage at -20 °C. A negative control containing all reaction components except the enzyme was included and analyzed by real-time PCR to exclude the possibility of DNA contamination in the RNA samples.

For real-time PCR, 1/40 of the reverse transcription product was used as a template. The reactions were performed in a C1000 thermocycler fitted with a CFX96 real-time system (Bio-Rad Laboratories) with 300 nM of each corresponding primer pair and iQ SYBR Green Supermix (Bio-Rad). The specific primers used were LIR1-RT-F and LIR1-RT-R for *L*. *amazonensis LIR1*, UbH-RT-F and UbH-RT-R for *ubiquitin hydrolase* (*UbH*), and EGFP-LIR1-RT-F and EGFP-LIR1-RT-R for ectopically expressed *GFP-LIR1* quantification (S1 Table). The PCR reaction consisted of an initial denaturation step of 95 °C for 3 min followed by 40 cycles of 15 s at 95 °C and 30 s at 60 °C. The target gene expression levels were quantified according to a standard curve prepared from a ten-fold serial dilution of a quantified and linearized plasmid containing the DNA segment to be amplified.

For protein level determinations, total lysates of *L*. *amazonensis* expressing GFP-tagged LIR1 (plasma membrane) or EGFP (cytosol) were used. Briefly, for detection of GFP-tagged LIR1, 5x10^7^ parasites were lysed at room temperature in 100 μl of Thorner lysis buffer (50 mM Tris-HCl pH 6.8, 8 M urea, 5% SDS, 0.1 mM EDTA, 0.01% bromophenol blue, 5% β-mercaptoethanol). For detection of cytosolic EGFP, 5x10^7^ parasites were lysed in 100 μl of lysis buffer containing 1% Triton, 150 mM NaCl, 50 mM Tris-HCl pH 7.6 and a protease inhibitor cocktail (Roche), lysates were clarified at 14,000 g for 15 min at 4°C, SDS sample buffer was added to the supernatant and samples were boiled for 5 min. 20 μg of each lysate sample were subjected to Western blot analysis using a rabbit polyclonal anti-GFP (A-11122, ThermoFisher Scientific) and rabbit polyclonal anti-arginase antibodies [34] as loading control. Blots were developed using Clarity Western blot ECL substrate (Bio-Rad) and detected with a Fuji LAS-3000 Imaging System and the Image Reader LAS-3000 software (Fuji). Digital quantifications of chemiluminescence were performed using NIH ImageJ 1.50i software.

### Generation of *L*. *amazonensis LIR1* overexpressor, knockout and complemented cell lines

To generate parasites expressing LIR1 tagged with triple-Flag (3xFlag), the gene ORF was amplified using the primers LIR1-Flag-N and ORF-R for N-terminal fusion, and ORF-F and LIR1-Flag-C for C-terminal fusion (S1 Table). The amplicons were purified and cloned into the *Leishmania* expression vector pXGHyg [49] generating the vectors p-3xFlag-LIR1 and p-LIR1-3xFlag. For generating parasites expressing LIR1 tagged with GFP, the gene ORF was amplified using the primers LIR1-GFP-N-F and LIR1-GFP-N-R for N-terminal fusion, and LIR1-GFP-C-F and LIR1-GFP-C-R for C-terminal (S1 Table). The amplicons were purified and cloned into the *Bam*HI site of the *Leishmania* expression vectors pXG-GFP2+ and pXG-GFP+ [49], for GFP fusion at the N-(p-GFP-LIR1) and C-(p-LIR1-GFP) terminal respectively. For generating parasites expressing cytosolic EGFP, we utilized the pXG-EGFP plasmid provided by Lucile Maria Floeter-Winter (University of São Paulo) as previously described [50]. All constructs were transfected by electroporation [51]. Isolated clones were selected in M199 containing 30 μg/ml Hygromycin for the 3x-Flag tagged constructs, or 20 μg/ml G418 for the GFP-LIR1 and EGFP constructs.

The gene deletion constructs were based on the drug resistance cassette donor plasmids pCR-DRC and backbone plasmid pBB-CmR-ccdB (courtesy of Phillip Yates) [52]. *LIR1* 5’ and 3’ UTR flanking cassettes were obtained using the following primers containing *Sfi*I restriction sites: 5’SfiI-A-F, 5’SfiI-B-R, 3’SfiI-C-F and 3’SfiI-D-R (S1 Table). These 5’ and 3’ UTR amplicons were cloned into the backbone plasmid pBB-CmR-ccdB flanking the drug resistance cassettes from pCR-NEO and pCR-BSD. The linearized resulting constructs were transfected by electroporation in 2 independent rounds [51]. Single knockout (SKO) clones from the first electroporation round were selected in M199 containing 20 μg/ml Neomycin. Selected SKO clones were subjected to a second electroporation round with a distinct drug resistance cassette and then double knockouts (KO) clones were selected in M199 containing 20 μg/ml Neomycin plus 20 μg/ml Blasticidin. To generate complemented cell lines, selected double knockout clones were transfected with the linearized construct pIR1-LIR1 (AB) or the plasmid pXG-LIR1 (AB pXG). These constructs were obtained cloning the *LIR1* ORF into the pIR1SAT plasmid designed for integration into the SSU rRNA locus [51] or into the pXGHyg plasmid for LIR1 ectopic expression [48]. Add-back clones were selected in M199 containing 20 μg/ml Neomycin, 20 μg/ml Blasticidin and 50 μg/ml Nourseothricin (AB) or 30 μg/ml Hygromycin (AB pX).

To confirm replacement of the *LIR1* ORF by the drug resistance cassettes or integration into the SSU rRNA locus, genomic DNA from the selected clones was extracted using the NucleoSpin Tissue kit (Macherey-Nagel GmbH & Co. KG) following manufacturers’ instructions. The resulting DNA samples were used as template in PCR amplification analyses (S3 Fig).

### *Leishmania* immunofluorescence microscopy

Promastigotes were fixed with 4% paraformaldehyde and attached to poly L-lysine coated slides (multitest 8-well; MP Biomedicals). For immunofluorescence of intracellular amastigotes, coverslips with BMM infected as described below were used. The fixed cells were quenched with 50 mM NH_4_Cl for 1 h and permeabilized with PBS containing 0.1% Triton for 15 min, prior to blocking with PBS containing 5% horse serum and 1% bovine serum albumin (BSA) for 1 h at room temperature. For 3xFLAG tag detection, mouse anti-FLAG (F1804, Sigma) was used as primary antibody (1:500 dilution in PBS-1% BSA) for 1 h, followed by anti-mouse IgG AlexaFluor 488 (1:5000 dilution) as secondary antibody. For Lamp-1 detection, fixed infected BMM were quenched with 50 mM NH_4_Cl for 1 h, blocked with PBS containing 3% BSA, permeabilized with PBS containing 0.15% saponin for 15 min, followed by incubation with anti-Lamp1 (1D4B) (Abcam) diluted 1:100 in PBS followed by anti-rat IgG AlexaFluor 594 (1:1000 dilution) as secondary antibody. All samples were incubated with 1 μg/ml DAPI for nuclear staining. Slides were mounted with ProLong Gold antifade reagent (Invitrogen). Images were acquired through a Deltavision Elite Deconvolution microscope (GE Healthcare) and processed using Volocity Suite (PerkinElmer).

### Generation and analysis of transgenic *Arabidopsis thaliana*

The *LIR1* coding sequence was amplified with the primers ORF-F and ORF-R and cloned into the pCR/8/GW/TOPO vector (Invitrogen). After sequence verification, the fragment was recombined into pEarleyGate 103 [53] to generate a LIR1-GFP fusion construct under the 35S promoter (a constitutive promoter from Cauliflower Mosaic Virus). The construct was introduced into *Arabidopsis thaliana* wild type (ecotype Ler) and *irt-1-1* mutants (ecotype Wassilewskija) [31] via *Agrobacterium* by floral dipping [54]. The *irt1-1* mutant was kindly provided by Dr. Catherine Curie (CNRS Montpellier).

Plants were grown on Metromix soil (Griffin) under a 16 h light-8 h dark cycle at 25 °C. When plants were grown in soil, iron was supplied as Sequestrene (Sequestrene 330 Fe Chelate, Basf) water solution 0.5 g/L, equivalent to about 500 μM Fe-EDDHA (Sigma Aldrich). When plants were grown on media plates, surface sterilized seeds were germinated on half-strength Murashige and Skoog medium containing 0.7% (w/v) phyto agar (Research Products International). Iron was supplied as 50 μM NaFe-EDTA (ethylenediaminetetraacetic acid ferric sodium salt, Sigma Aldrich) in growth media.

Mesophyll protoplasts of transgenic plants were prepared as previously described [55].

Imaging of protoplasts and whole roots of transgenic plants was carried out using a Leica SPX5 confocal microscope. The root cell membrane was stained with 10 μg/ml propidium iodide (PI), and the protoplast membrane was stained with 20 μM FM4-64 (Molecular Probes) for 5 min.

### ^55^Fe uptake and efflux

For uptake assays, mid to end-log phase promastigotes were washed once with OptiMEM (Gibco) and incubated for 20 min at 26 °C in OptiMEM contaning 50 μM of ascorbic acid to a final concentration of 1.25 x 10^8^ parasites/ml. To initiate uptake 1 μM ^55^FeCl in 1 μM ascorbate (^55^Fe ascorbate) was added to the cell suspension and samples were incubated at 26 °C or ice for various time intervals. At the end of the incubation period, 400 μl (5x10^7^ cells) were transferred to 1.5 ml tubes on ice containing 1 ml of quench buffer (0.1 M Tris HCl pH 7.4, 0.1 M Succinate, 10 mM EDTA). The cells were collected, washed twice with quench buffer and once with HBSS, and lysed with 100 μl of 50 mM NaOH followed by addition of 100 μl 50 mM HCl.

For efflux assays, mid to end-log phase promastigotes were incubated in heme-depleted medium plus 50 μM deferoxamine (DFO). After 18 h, the cells were washed 3 times with HBSS (Gibco) and incubated for 20 min at 26 °C in HBSS containing 50 μM ascorbic acid to a final concentration of 1.25 x 10^8^ parasites/ml. To initiate uptake 1 μM ^55^FeCl in 1 μM ascorbate (^55^Fe ascorbate) was added to the cell suspension and samples were incubated at 26 °C or ice for 90 min. At the end of the uptake period cells were washed 3 times with cold HBSS. To start the chase period cells were transferred to HBSS containing 50 μM ascorbic acid and 100 μM of “cold” iron (C_6_H_8_O_7_Fe.H_3_N—ammonium iron (III) citrate) and incubated at 26 °C or ice for various time intervals. At the end of the incubation period 400 μl (5x10^7^ cells) were transferred to 1.5 ml tubes on ice, collected and lysed with 100 μl of 50 mM NaOH followed by addition of 100 μl 50 mM HCl. The radioactivity in the lysates was determined by liquid scintillation counting. ^55^Fe levels were normalized to the sample protein content, determined using a Pierce BCA protein assay kit (Thermo Scientific).

### Elemental iron quantification

Inductively coupled plasma mass spectrometry (ICP-MS) analysis was performed as previously described [56]. Briefly, the concentrations of two Fe isotopes (^56^Fe, ^57^Fe) (μg) were quantified using a final internal standard concentration of 50 μg/L Ga and normalized per gram of total protein content of digested promastigote cells or plant tissues. 10^8^ *L*. *amazonensis* late log promastigotes were digested for 1 h in 100 μl of HNO_3_ 70%. 100 mg of *Arabidopsis thaliana* leaves were digested with 400 μl of 50% HNO_3_/50% H_2_O_2_ overnight at 50 °C, after drying overnight at 60 °C. Lysates were diluted 10 fold into 1% nitric acid for the analysis. Two isotopes were measured for Fe (^56^Fe, ^57^Fe) and Zn (^64^Zn, ^66^Zn). Each sample was subjected to two (promastigote samples) or three (plant samples) independent injections for the ICP-MS run.

### *In vitro* mouse macrophage infections

Mouse bone marrow macrophages (BMM) were prepared from C57BL/6 mice (Jackson Laboratories) as previously described [57]. A total of 10^6^ BMMs per well were plated on glass coverslips in 6-well plates and incubated for 24 h at 37 °C 5% CO_2_ in BMM media: RPMI 1640 containing L-glutamine medium (Gibco) supplemented with 20% endotoxin-free FBS (Gibco), 5% penicillin/streptomycin, 132 μg/ml Na pyruvate, 50 ng/ml human macrophage colony-stimulating factor (M-CSF) (PeproTech). Infective metacyclic forms were purified from stationary phase promastigote cultures (second to third day after entering stationary phase) using the 3A.1 monoclonal antibody as described [40]. Purified metacyclics were added at a ratio of 3 parasites per macrophage for 3 h at 34 °C. The cells were washed 3 times with PBS and fixed or incubated in BMM media to complete 24, 48, 72 and 96 h of infection. Coverslips were fixed in 4% paraformaldehyde and incubated with 1 μg/ml DAPI (4’,6-diamidino-2-phenylindole) for 1 h, after permeabilization with 0.1% Triton X-100 for 10 min. The number of intracellular parasites was determined by counting the total macrophages and the total intracellular parasites per microscopic field (Nikon E200 epifluorescence microscope). At least 200 host cells, in triplicate, were analyzed for each time point.

### Viability of metacyclic forms

Cell viability was assessed using AlamarBlue Cell Viability Reagent (Invitrogen) following the manufacturers’ instructions. Briefly, 20 μl of AlamarBlue was added to 1, 2 and 4 x 10^6^ purified metacyclics in 200 μl M199. After 4 h fluorescence levels were determined using a fluorescence plate reader (SpectraMax M5, Molecular Devices) at 550 nm excitation and 590 nm emission.

### *In vivo* infection and parasite tissue load determination

Six-week-old female C57BL/6 mice (Jackson Laboratories) (n = 5 per group) were inoculated with 10^6^ purified metacyclics from *L*. *amazonensis* WT, *LIR1* SKO, KO and add-back (AB) in the left hind footpad in a volume of 0.05 ml. Lesion progression was monitored once a week by measuring the difference in thickness between the left and right hind footpads with a caliper (Mitutoyo Corp., Japan). The parasite load in the infected tissue was determined after 10 weeks in the infected tissue collected from footpad lesions of sacrificed mice by limiting dilution [38].

### Statistical analysis

Data were analyzed by an unpaired two-tailed Student’s t test using GraphPad Prism software. The data meet the assumptions of the test. Variance is similar among compared groups. A result was considered significant at a p value < 0.05. P values and the number of times each experiment was repeated are stated in the figure legends. No statistical method was used to predetermine sample size.

### Ethics statement

All animal work was conducted in accordance with the guidelines provided by National Institutes of Health for housing and care of the laboratory animals and performed under protocol # R-14-79 approved by the University of Maryland College Park Institutional Animal Care and Use Committee on January 11, 2018. The University of Maryland at College Park is an AAALAC-accredited institution.

## Supporting information

S1 Table Oligonucleotides used in this study. (DOCX)

S1 Fig Modulation of *LIR1* transcript levels by iron availability. (a) Endogenous *LIR1* transcript levels were determined and normalized by *UbH* transcript levels in *L*. *amazonensis* treated for 24 h in regular promastigote growth medium (C), heme-depleted medium (HD), heme-depleted medium plus 50 μM DFO (HD+DFO) or iron-depleted medium (ID). The graph shows the individual values of *LIR1*/*UbH* transcript levels and means of duplicate experiments. (b) *LIR1* transcript levels were determined using primers within the *LIR1* ORF (detecting both endogenous and ectopically expressed *LIR1*) or a forward primer within the *GFP* sequence and a reverse primer within the *LIR1* ORF (detecting only ectopically expressed *GFP-LIR1*), and normalized by *UbH* transcript levels, in promastigotes ectopically expressing *GFP-LIR1* (p-GFP-LIR1) and grown under iron depletion (HD, heme depletion; HD+DFO, heme depletion plus the iron chelator DFO) or iron supplementation (0.5 Fe, 0.5 mM FeSO_4;_ 1 Fe, 1 mM FeSO_4_). The data show the mean ± SEM of *GFP-LIR1*/*UbH* transcript levels normalized by the respective controls in 3 independent experiments. * p = 0.048 (1 vs 0 Fe); *** p = 0.0007 (1 vs 0 Fe).
(TIF)

S2 Fig LIR1 expression in *Arabidopsis thaliana*. (a) Confocal images of leaf mesophyll protoplasts isolated from transgenic *Arabidopsis thaliana* expressing *LIR1* (35S::LIR1-GFP-His). Green, GFP. Red, cell wall/membrane stained with propidium iodide (PI, top panels) or FM4-64 (lower panels). Scale bars: 25 μm. (b) Phenotype of 6 weeks-old wild type (WT), *irt1-/-* and 6 transgenic lines of WT *Arabidopsis thaliana* expressing *LIR1* (35S::LIR1) (A-C), or *LIR1* fused to GFP (35S::LIR1-GFP) (D-F) grown in soil and irrigated with water.
(TIF)

S3 Fig Generation of *L*. *amazonensis LIR1* mutants. (a) Gene replacement strategy. The targeting fragments are shown below the *LIR1* chromosomal locus. The arrows indicate the position of oligonucleotide primers used for PCR confirmation of the insertions. (b) Strategy for insertion into the SSU rRNA locus. The targeting fragment containing *LIR1* ORF is shown below the SSU rRNA locus. The arrows indicate the position of oligonucleotide primers used for PCR confirmation of the insertions. (c) Agarose gel images showing the amplicons obtained using as template DNA from wild type (WT), *LIR1* single knockout clones (SKO_1-3_), *LIR1* double knockout clone (KO), add-back (AB) *L*. *amazonensis* clones, and water as the PCR negative control (-). The brackets below the gels indicate the pair of oligonucleotide primers used for each sample. (d) *LIR1* transcript levels were determined by real time PCR and normalized by *UbH* transcript levels for wild type (WT), *LIR1* single knockout (SKO), *LIR1* double knockout (KO) and add-back (AB) *L*. *amazonensis* clones. The graph shows the individual values and means of duplicate experiments.
(TIF)

S4 Fig Impact of LIR1 deficiency and add-back on promastigote replication: Effect of excess iron and growth curves of independent LIR1 deficient and add-back clones. (a) Change in promastigote numbers of wild type (WT), *LIR1* single knockout (SKO), *LIR1* double knockout (KO) and add-back (AB) *L*. *amazonensis* grown in medium containing extra iron (0.5 mM FeSO_4_) relative to growth in regular medium (control). The data are expressed as the parasite numbers/ml in medium containing extra iron subtracted from the parasite numbers/ml in regular medium. Each data point represents the mean ± SEM of 3 independent experiments. ** p = 0.0073 (KO vs. WT). (b) Growth curves in regular growth medium of wild type (WT), 2 independent clones of *LIR1* single knockout (SKO a and b), and 2 independent clones of *LIR1* double knockout (KO a and b). The values correspond to the average of triplicate determinations. (c) Growth curves in regular growth medium of wild type (WT), 2 independent clones of *LIR1* single knockout (SKO a and b), *LIR1* double knockout (KO), *LIR1* SSU knock-in add-back (AB) and an add-back ectopically expressing *LIR1* (AB pX). The values correspond to the average of triplicate determinations.
(TIF)

S5 Fig Infectivity of independent LIR1 deficient and add-back clones. Microscopic quantification of intracellular parasites in BMM infected with metacyclic forms (multiplicity of infection = 3). (a) *L*. *amazonensis* strains: wild type (WT), 2 independent clones of *LIR1* double knockout (KO a and b), and 2 independent clones of *LIR1* single knockout (SKO a and b). The values represent the mean ± SEM of triplicate determinations. ** p = 0.002 (KO a and b vs. WT 48 h); ** p = 0.008 (KO b vs SKO b 48 h). (b) *L*. *amazonensis* strains: wild type (WT), *LIR1* double knockout (KO), *LIR1* single knockout (SKO), the *LIR1* SSU knock-in add-back (AB) and the add-back ectopically expressing *LIR1* (AB pX). The values represent the mean ± SEM of triplicate determinations. * p = 0.0308 (KO vs. AB 24 h); * p = 0.0383 (KO vs. AB pX 24 h); *** p = 0.0002 (KO vs. WT 48 h); * p = 0.0376 (KO vs. SKO 48 h); *** p = 0.0007 (KO vs. AB 48 h); **** p < 0.0001 (KO vs. AB pX 48 h and 72 h); * p = 0.0108 (KO vs. SKO 72 h); ** p = 0.0063 (KO vs. AB 72 h).
(TIF)
