# Supplementary material for: A MFS-like plasma membrane transporter required for Leishmania virulence protects the parasites from iron toxicity
Source: PLoS Pathog. 2018 Jun 15;14(6):e1007140. doi: 10.1371/journal.ppat.1007140 (PMC6021107; doi:10.1371/journal.ppat.1007140)
Supplement: S1 Table — (DOCX) [file ppat.1007140.s001.docx]

**S1 Table.** Oligonucleotides used in this study.

| Primer | Sequence (5´ - 3´) |
| --- | --- |
| LIR1-RT-F | GCCAGCTTCGTCATTGTATTG |
| LIR1-RT-R | CTCGTAGTAGGTGACGGTAGA |
| UbH-RT-F | AACGTGAACAACTGGATGTGCGTC |
| UbH-RT-R | ATGGTACCAAGCTTGACACATGCC |
| EGFP-LIR1-RT-F | CATGGACGAGCTGTACAAGA |
| EGFP-LIR1-RT-R | TGCGAGAAGATGTGGCTT |
| LIR1-Flag-N | ATGGACTACAAAGACCATGACGGTGATTATAAAGATCATGACATC  GATTACAAGGACGACGATGACAAGTCTTCGCAGGGCTCAGC |
| LIR1-Flag-C | TTACTTGTCATCGTCGTCCTTGTAATCGATGTCATGATCTTTATAAT  CACCGTCATGGTCTTTGTAGTCTTGCTCAAGGACGAGACC |
| ORF-F | ATGTCTTCGCAGGGCTC |
| ORF-R | TTATTGCTCAAGGACGAGACCC |
| LIR1-GFP-N-F | GGATCCTCTTCGCAGGGCTCAGC |
| LIR1-GFP-N-R | GGATCCTTATTGCTCAAGGACGAGACCC |
| LIR1-GFP-C-F | GGATCCATGTCTTCGCAGGGCTC |
| LIR1-GFP-C-R | GGATCCTTGCTCAAGGACGAGACCC |
| 5’SfiI-A-F | GAGGCCACCTAGGCCTGCATTCTCTCCTCTTCTTTGG |
| 5’SfiI-B-R | GAGGCCACGCAGGCCGGTGGTTTGCTTATTGCGTATC |
| 3’SfiI-C-F | GAGGCCTCTGTGGCCTAACGAAGGAAATGTGAAGCCTC |
| 3’SfiI-D-R | GAGGCCTGACTGGCCCGAAACACAGGCCAACATC |
| 5’UTR-F | GTGTGTAAGCGTACGTGGTT |
| 3’UTR-F | GTGCACGTCTCTGAAAGCA |
| BSD-F | TCGCGATCGGAAATGAGAAC |
| BSD-R | CTGTCCTTCACTATCGCTTTGA |
| S1-SSU | GATCTGGTTGATTCTGCCAG |
