# Supplementary figures and images for: A MFS-like plasma membrane transporter required for Leishmania virulence protects the parasites from iron toxicity
Source: PLoS Pathog. 2018 Jun 15;14(6):e1007140. doi: 10.1371/journal.ppat.1007140 (PMC6021107; doi:10.1371/journal.ppat.1007140)

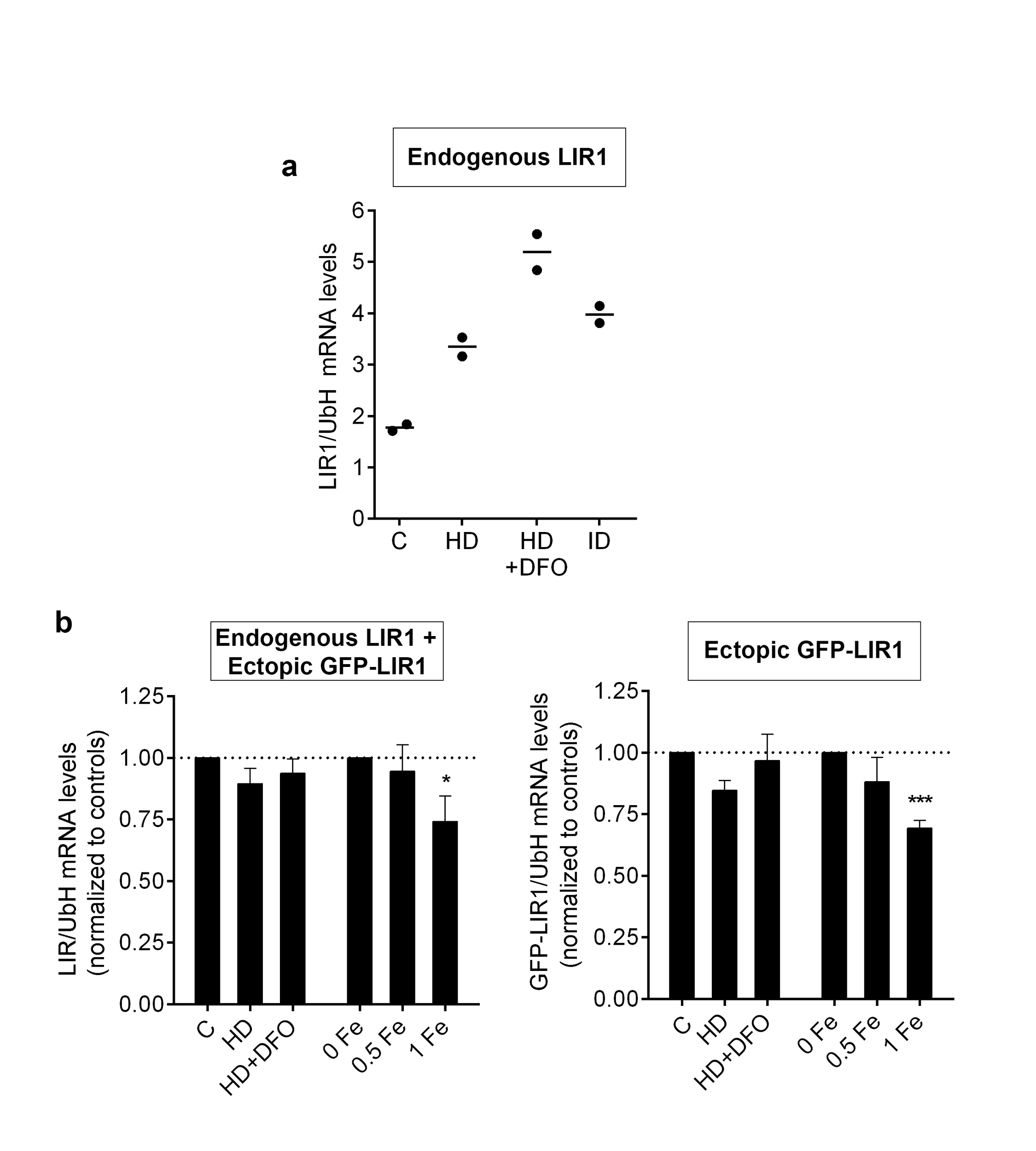

Supplement: S1 Fig — (a) Endogenous LIR1 transcript levels were determined and normalized by UbH transcript levels in L. amazonensis treated for 24 h in regular promastigote growth medium (C), heme-depleted medium (HD), heme-depleted medium plus 50 μM DFO (HD+DFO) or iron-depleted medium (ID). The graph shows the individual values of LIR1/UbH transcript levels and means of duplicate experiments. (b) LIR1 transcript levels were determined using primers within the LIR1 ORF (detecting both endogenous and ectopically expressed LIR1) or a forward primer within the GFP sequence and a reverse primer within the LIR1 ORF (detecting only ectopically expressed GFP-LIR1), and normalized by UbH transcript levels, in promastigotes ectopically expressing GFP-LIR1 (p-GFP-LIR1) and grown under iron depletion (HD, heme depletion; HD+DFO, heme depletion plus the iron chelator DFO) or iron supplementation (0.5 Fe, 0.5 mM FeSO4; 1 Fe, 1 mM FeSO4). The data show the mean ± SEM of GFP-LIR1/UbH transcript levels normalized by the respective controls in 3 independent experiments. * p = 0.048 (1 vs 0 Fe); *** p = 0.0007 (1 vs 0 Fe). (TIF) [file ppat.1007140.s002.tif]

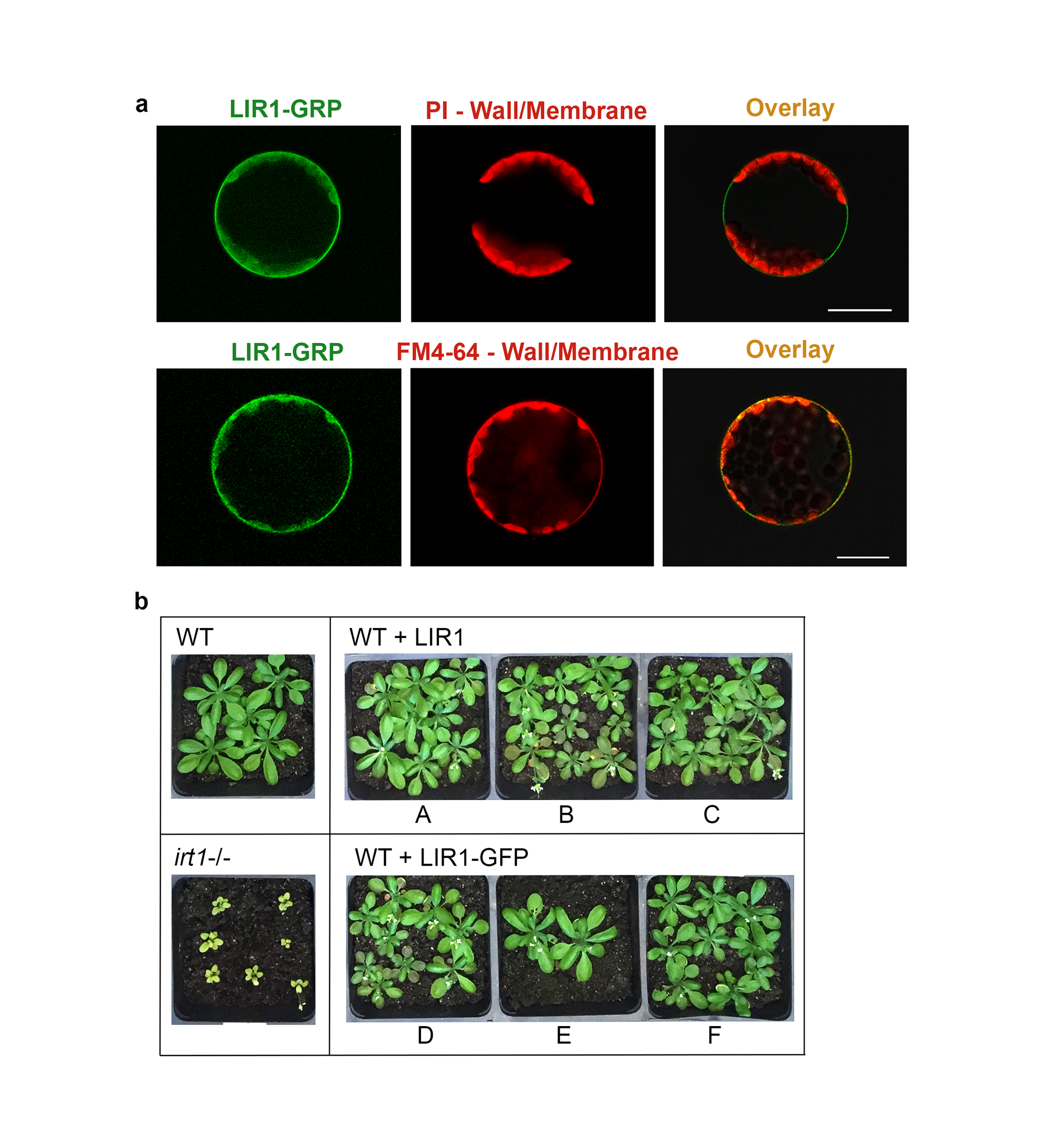

Supplement: S2 Fig — (a) Confocal images of leaf mesophyll protoplasts isolated from transgenic Arabidopsis thaliana expressing LIR1 (35S::LIR1-GFP-His). Green, GFP. Red, cell wall/membrane stained with propidium iodide (PI, top panels) or FM4-64 (lower panels). Scale bars: 25 μm. (b) Phenotype of 6 weeks-old wild type (WT), irt1-/- and 6 transgenic lines of WT Arabidopsis thaliana expressing LIR1 (35S::LIR1) (A-C), or LIR1 fused to GFP (35S::LIR1-GFP) (D-F) grown in soil and irrigated with water. (TIF) [file ppat.1007140.s003.tif]

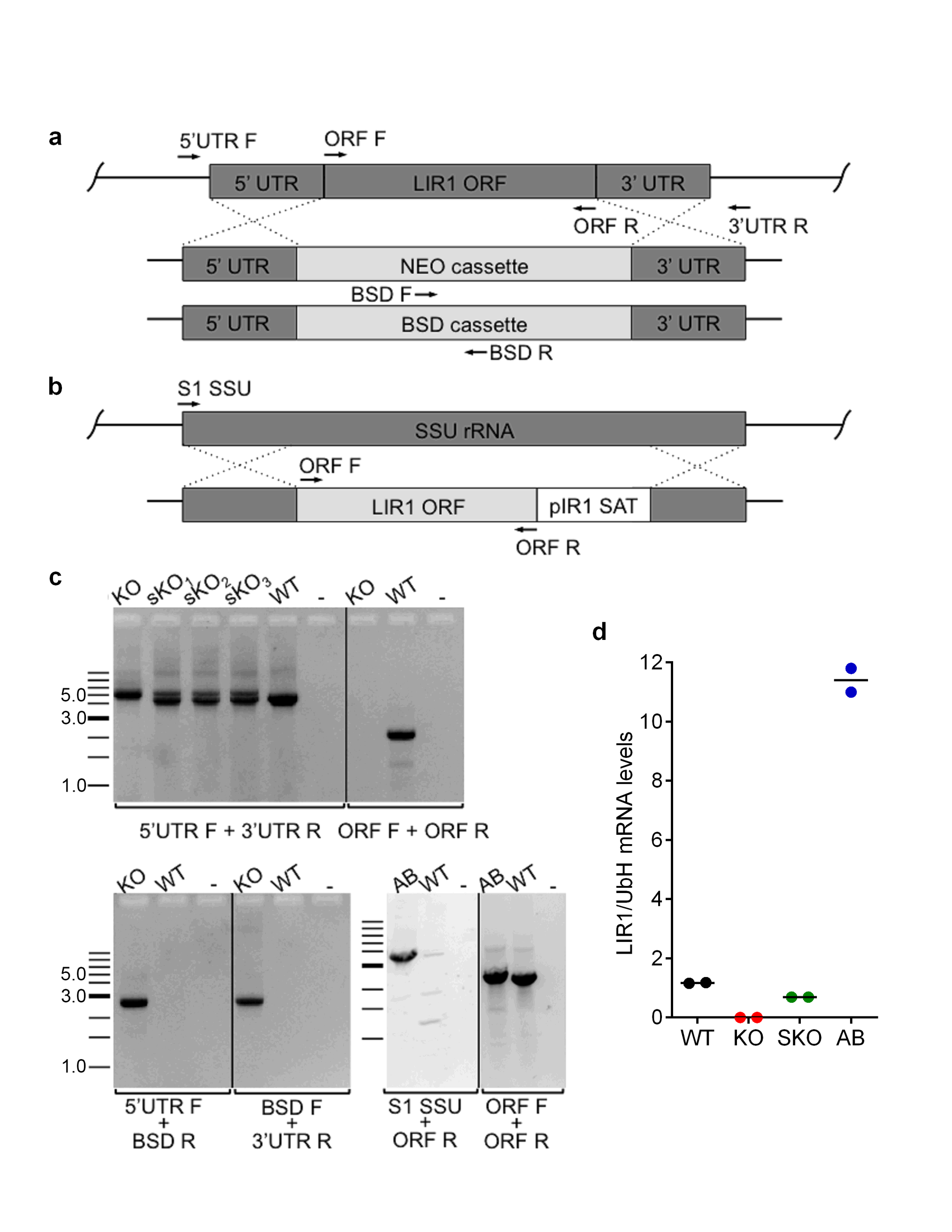

Supplement: S3 Fig — (a) Gene replacement strategy. The targeting fragments are shown below the LIR1 chromosomal locus. The arrows indicate the position of oligonucleotide primers used for PCR confirmation of the insertions. (b) Strategy for insertion into the SSU rRNA locus. The targeting fragment containing LIR1 ORF is shown below the SSU rRNA locus. The arrows indicate the position of oligonucleotide primers used for PCR confirmation of the insertions. (c) Agarose gel images showing the amplicons obtained using as template DNA from wild type (WT), LIR1 single knockout clones (SKO1-3), LIR1 double knockout clone (KO), add-back (AB) L. amazonensis clones, and water as the PCR negative control (-). The brackets below the gels indicate the pair of oligonucleotide primers used for each sample. (d) LIR1 transcript levels were determined by real time PCR and normalized by UbH transcript levels for wild type (WT), LIR1 single knockout (SKO), LIR1 double knockout (KO) and add-back (AB) L. amazonensis clones. The graph shows the individual values and means of duplicate experiments. (TIF) [file ppat.1007140.s004.tif]

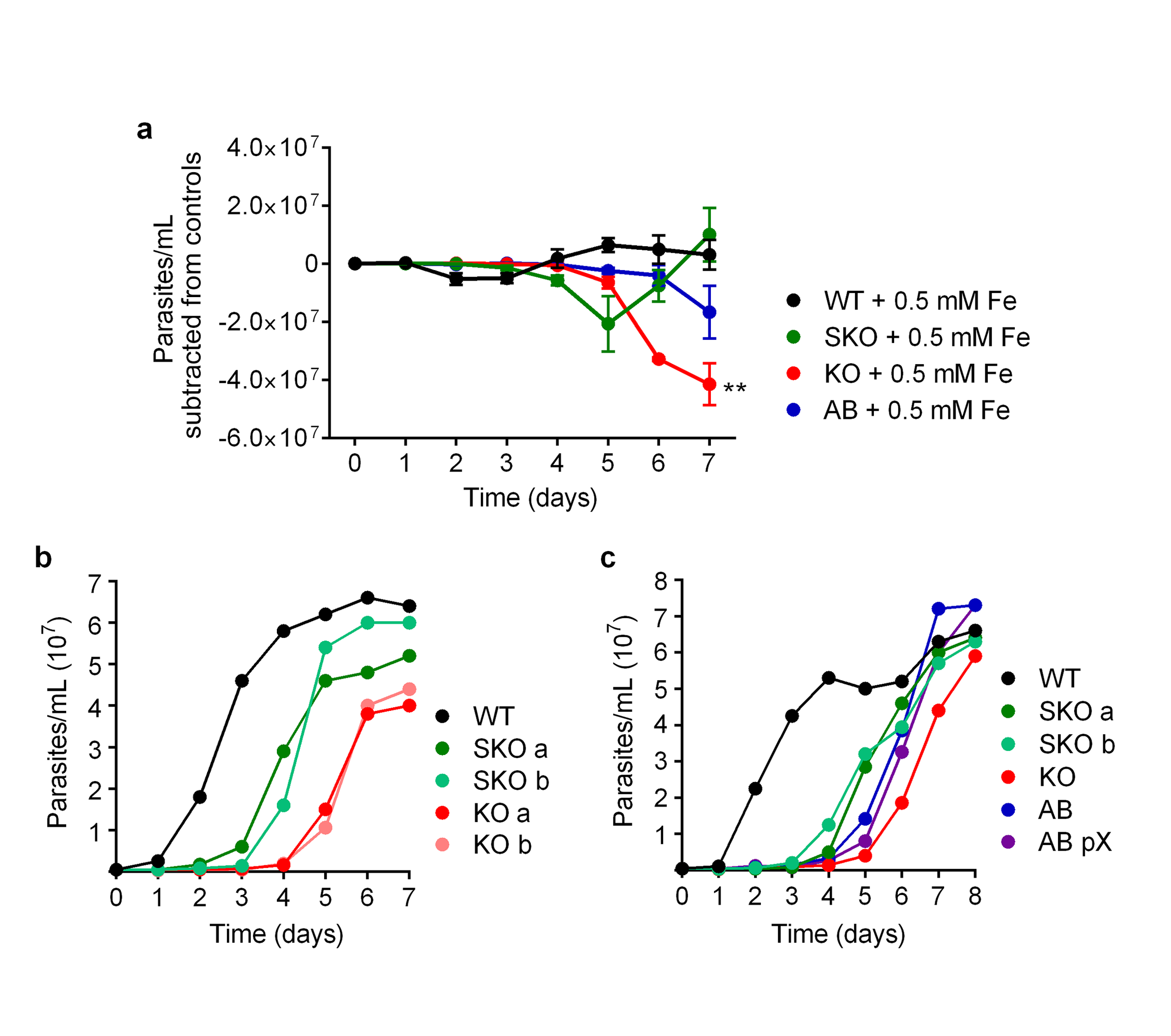

Supplement: S4 Fig — (a) Change in promastigote numbers of wild type (WT), LIR1 single knockout (SKO), LIR1 double knockout (KO) and add-back (AB) L. amazonensis grown in medium containing extra iron (0.5 mM FeSO4) relative to growth in regular medium (control). The data are expressed as the parasite numbers/ml in medium containing extra iron subtracted from the parasite numbers/ml in regular medium. Each data point represents the mean ± SEM of 3 independent experiments. ** p = 0.0073 (KO vs. WT). (b) Growth curves in regular growth medium of wild type (WT), 2 independent clones of LIR1 single knockout (SKO a and b), and 2 independent clones of LIR1 double knockout (KO a and b). The values correspond to the average of triplicate determinations. (c) Growth curves in regular growth medium of wild type (WT), 2 independent clones of LIR1 single knockout (SKO a and b), LIR1 double knockout (KO), LIR1 SSU knock-in add-back (AB) and an add-back ectopically expressing LIR1 (AB pX). The values correspond to the average of triplicate determinations. (TIF) [file ppat.1007140.s005.tif]

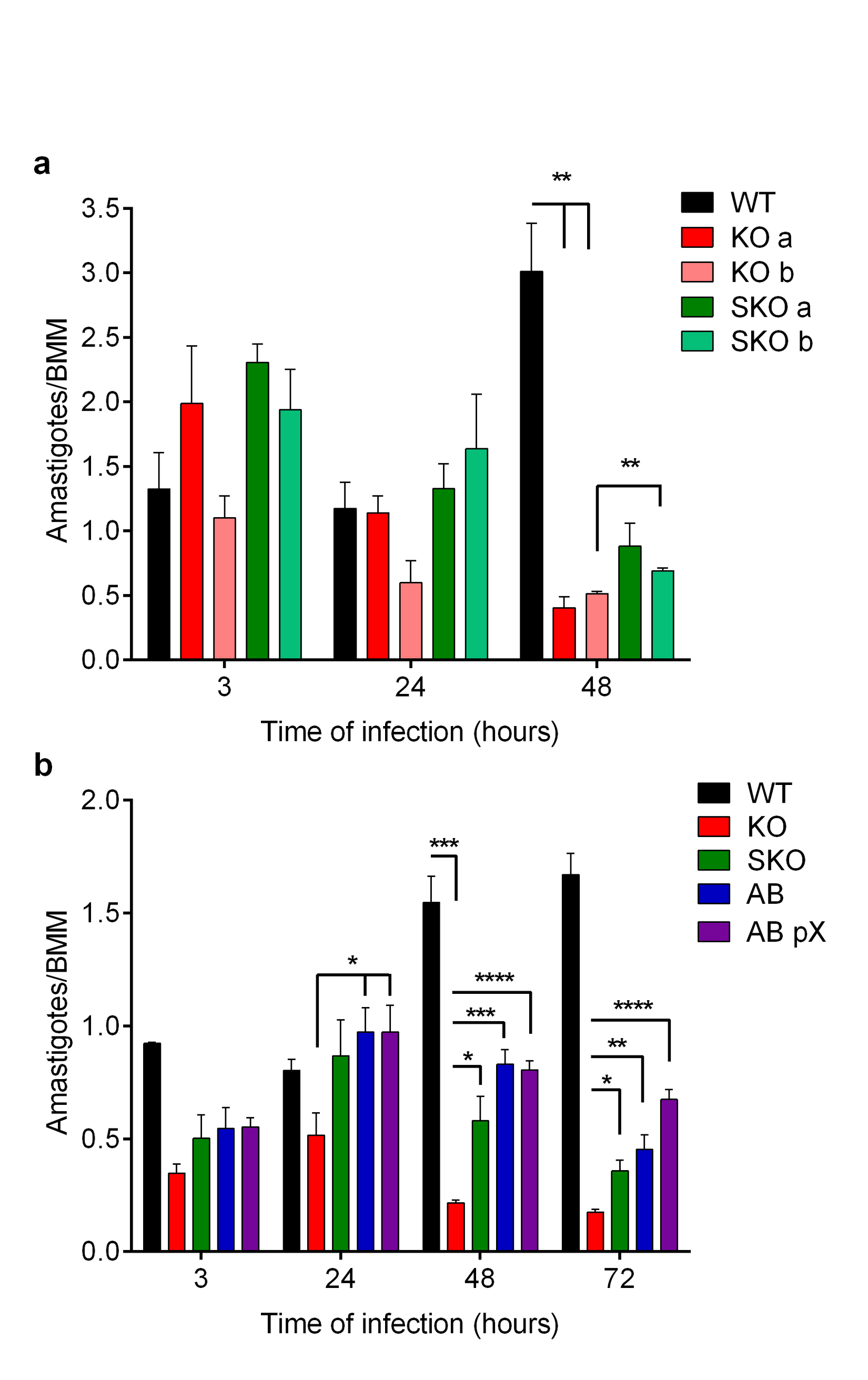

Supplement: S5 Fig — Microscopic quantification of intracellular parasites in BMM infected with metacyclic forms (multiplicity of infection = 3). (a) L. amazonensis strains: wild type (WT), 2 independent clones of LIR1 double knockout (KO a and b), and 2 independent clones of LIR1 single knockout (SKO a and b). The values represent the mean ± SEM of triplicate determinations. ** p = 0.002 (KO a and b vs. WT 48 h); ** p = 0.008 (KO b vs SKO b 48 h). (b) L. amazonensis strains: wild type (WT), LIR1 double knockout (KO), LIR1 single knockout (SKO), the LIR1 SSU knock-in add-back (AB) and the add-back ectopically expressing LIR1 (AB pX). The values represent the mean ± SEM of triplicate determinations. * p = 0.0308 (KO vs. AB 24 h); * p = 0.0383 (KO vs. AB pX 24 h); *** p = 0.0002 (KO vs. WT 48 h); * p = 0.0376 (KO vs. SKO 48 h); *** p = 0.0007 (KO vs. AB 48 h); **** p < 0.0001 (KO vs. AB pX 48 h and 72 h); * p = 0.0108 (KO vs. SKO 72 h); ** p = 0.0063 (KO vs. AB 72 h). (TIF) [file ppat.1007140.s006.tif]
